# Integrating on-farm and genomic information improves the predictive ability of milk infrared prediction of blood indicators of metabolic disorders in dairy cows

**DOI:** 10.1186/s12711-023-00795-1

**Published:** 2023-04-03

**Authors:** Lucio F. M. Mota, Diana Giannuzzi, Sara Pegolo, Erminio Trevisi, Paolo Ajmone-Marsan, Alessio Cecchinato

**Affiliations:** 1 Department of Agronomy, Food, Natural Resources, Animals and Environment (DAFNAE), University of Padova, 35020 Legnaro, PD Italy; 2 Department of Animal Science, Food and Nutrition (DIANA) and the Romeo and Enrica Invernizzi Research Center for Sustainable Dairy Production (CREI), Faculty of Agricultural, Food, and Environmental Sciences, Università Cattolica del Sacro Cuore, 29122 Piacenza, Italy; 3 Nutrigenomics and Proteomics Research Center, Università Cattolica del Sacro Cuore, 29122 Piacenza, Italy

## Abstract

**Background:**

Blood metabolic profiles can be used to assess metabolic disorders and to evaluate the health status of dairy cows. Given that these analyses are time-consuming, expensive, and stressful for the cows, there has been increased interest in Fourier transform infrared (FTIR) spectroscopy of milk samples as a rapid, cost-effective alternative for predicting metabolic disturbances. The integration of FTIR data with other layers of information such as genomic and on-farm data (days in milk (DIM) and parity) has been proposed to further enhance the predictive ability of statistical methods. Here, we developed a phenotype prediction approach for a panel of blood metabolites based on a combination of milk FTIR data, on-farm data, and genomic information recorded on 1150 Holstein cows, using BayesB and gradient boosting machine (GBM) models, with tenfold, batch-out and herd-out cross-validation (CV) scenarios.

**Results:**

The predictive ability of these approaches was measured by the coefficient of determination (R^2^). The results show that, compared to the model that includes only FTIR data, integration of both on-farm (DIM and parity) and genomic information with FTIR data improves the R^2^ for blood metabolites across the three CV scenarios, especially with the herd-out CV: R^2^ values ranged from 5.9 to 17.8% for BayesB, from 8.2 to 16.9% for GBM with the tenfold random CV, from 3.8 to 13.5% for BayesB and from 8.6 to 17.5% for GBM with the batch-out CV, and from 8.4 to 23.0% for BayesB and from 8.1 to 23.8% for GBM with the herd-out CV. Overall, with the model that includes the three sources of data, GBM was more accurate than BayesB with accuracies across the CV scenarios increasing by 7.1% for energy-related metabolites, 10.7% for liver function/hepatic damage, 9.6% for oxidative stress, 6.1% for inflammation/innate immunity, and 11.4% for mineral indicators.

**Conclusions:**

Our results show that, compared to using only milk FTIR data, a model integrating milk FTIR spectra with on-farm and genomic information improves the prediction of blood metabolic traits in Holstein cattle and that GBM is more accurate in predicting blood metabolites than BayesB, especially for the batch-out CV and herd-out CV scenarios.

**Supplementary Information:**

The online version contains supplementary material available at 10.1186/s12711-023-00795-1.

## Background

Dairy cows can experience a negative balance between energy intake and energy expenditure during the transition phase from late pregnancy to early lactation (i.e., three weeks before and after parturition), which leads to important metabolic challenges [1]. This condition increases the probability of metabolic stress, which results in an often complex interaction between catabolic and endocrine pathways that is caused by an increased imbalance between lipid mobilization and immune and hormonal status [2–4]. After calving, cows experience an increase in energy requirements for milk production and, when feed intake does not meet this high energy requirement, lipolysis is enhanced in the adipose tissue, which helps the cows to cope with these situations [2, 5]. However, this fat mobilization increases the concentrations of non-esterified fatty acids (NEFA) and β-hydroxybutyric acid (BHBA) in the bloodstream, which increase the risk of metabolic disorders, such as ketosis, hepatic lipidosis, liver damage and dysfunction [6], impaired hormone regulation causing hypocalcemia and hypomagnesemia [7, 8] and altered immune response [9]. The occurrence of metabolic disorders during early lactation has a profound negative effect on the profitability of the dairy cattle production system due to reduced milk production and reproductive performance and increased incidences of metritis and mastitis [10]. Furthermore, recent studies have highlighted that immune-metabolic changes can start during late lactation and the dry-off period and have long-term carryover effects during the post-calving period and following lactation [11, 12]. Hence, to evaluate the immune and metabolic variations during mid and late lactation phase, indicators that are associated with inflammatory status, oxidative stress and innate immunity are required.

Blood metabolic profiling is a well-established method for monitoring the major risk factors for metabolic disorders and nutritional imbalance in dairy cows. Several metabolites are evaluated such as glucose and BHBA for monitoring energy status, urea as an indicator of protein status, ceruloplasmin, total proteins and oxidative stress parameters, as indicators of inflammatory disease, and the enzymes aspartate aminotransferase (AST), γ-glutamyl transferase (GGT) and paraoxonase (PON) as a measure of hepatic overload [4, 13]. Blood mineral modifications measure homeostatic imbalance, with blood calcium being the major indicator of milk fever, and serum potassium and zinc being indicators of systemic inflammation and oxidation [14]. Although blood metabolite profiling is an accurate method for detecting metabolic disorders in dairy cows, its assessment on a large scale is expensive and time-consuming for dairy companies. However, Enjalbert et al. [15] have shown that the ketone concentrations in the milk and blood are highly correlated (from 0.66 to 0.96), which raises the possibility of predicting these concentrations using Fourier transform infrared (FTIR) milk spectra, as a rapid, non-invasive and cost-effective method. The use of milk FTIR spectra to assess the metabolic status of dairy cows on a large scale is a promising approach, given the interaction that exists between the metabolic status and milk compounds, mainly fat and protein [16]. De Roos et al. [17] investigated the use of milk FTIR data to predict ketone bodies in milk and obtained a predictive ability (R^2^) of 0.64, while Grelet et al. [18] reported R^2^ values ranging from 0.39 to 0.70 for blood glucose, IGF-1, NEFA, and BHBA.

Variations in fat, fatty acids, and protein contents in milk due to the mobilization of adipose tissue during a period of negative energy balance (NEB) may reflect the metabolic status of cows [19–21]. Gross et al. [22] reported that an energy deficiency and a milk fat/milk protein ratio above 1.35 could be useful signals of NEB in cows in early and mid-lactation. The ability of FTIR spectra to accurately predict fat and protein contents offers the possibility of using milk spectra to predict key metabolites in the blood that originate from the increased lipid mobilization after parturition, which leads to changes in milk composition, such as increased fat and decreased protein contents [23].

The main issue with using FTIR to predict blood metabolites at the farm level is how can the infrared wavelength data be transformed into information that can be used to identify changes in milk composition related to blood metabolites [24]. To solve this, attention has been directed towards exploring statistical approaches, such as machine learning (ML), that offer greater flexibility in modeling the complex associations between milk FTIR and the target blood metabolites, thus improving FTIR predictive ability [25–28]. This flexibility is combined with the ability to deal with correlated high-dimensional data and to capture possible non-linear associations between milk FTIR and the phenotypic value of the observed trait. Moreover, studies have indicated that predictive ability is further improved when milk FTIR information is integrated with on-farm data such as days in milk (DIM) and parity, and/or genomic information in the statistical models [25, 29]. Therefore, we hypothesized that, by combining different sources of information, it could be possible to better capture the complex biological signals that affect blood metabolites, which better explain the phenotypic variability and enhance FTIR-based predictions. Thus, our aim was to evaluate the potential usefulness of combining milk FTIR information with on-farm (DIM and parity) and genomic information for phenotype predictions of blood metabolites related to energy, liver function/hepatic damage, oxidative stress, and inflammation/innate immunity, and minerals in Holstein cattle.

## Methods

### Field data

The present study was carried out as part of the BENELAT project that aimed at developing short- and long-term interventions for improving animal welfare and efficiency, and the quality of dairy cattle production [30]. Milk samples were collected during the evening milking from 1150 Holstein cows that belonged to two herds in northern Italy (Emilia-Romagna region) and were managed under similar dairy production systems. The cows were housed mostly in sand-bedded free stalls and fed twice daily on total mixed rations (TMR) based on corn and sorghum silage supplemented with concentrates. Diets were formulated according to the nutritional requirements recommended by the NRC (Nutrient Requirements of Dairy Cattle) [31]. The cows were sampled once after a health check; animals with clinical mastitis or receiving medical treatment were excluded from the study, because these situations can lead to changes in milk spectra and affect the predictive ability of FTIR.

The handling procedures of the animals were approved by the ethical committee of the Organismo Preposto al Benessere Degli Animali (OPBA; Organization for Animal Welfare) of the Università Cattolica del Sacro Cuore (Piacenza, Italy) and by the Italian Ministry of Health (protocol number 510/2019-PR of 19/07/2019). The study also followed the ARRIVE (Animal Research: Reporting of In Vivo Experiments) guidelines.

### Blood and milk sampling

Milk and blood samples were collected in 16 batches across two herds (i.e., herd/date combinations): 14 batches in 2019 (963 cows for herd 1) and two batches in 2020 (90 cows for herd 1 and 97 for herd 2). The average values (± standard deviation (SD)) obtained from the evening milking were 32.58 ± 8.70 for milk yield (kg), 3.76 ± 0.69 for fat (%), and 3.42 ± 0.36 for protein (%). The cows had an average DIM of 187.71 ± 106.04 and an average parity of 1.98 ± 1.05. Individual milk samples (50 mL to which bronopol preservative was added) were maintained at 4 °C until laboratory analysis (within 24 h), then transferred to the laboratory of the Breeders’ Association of the Veneto Region (ARAV, Padua, Italy) for milk composition analysis (fat, protein, casein, lactose, and urea contents) using a Milkoscan FT6000 infrared analyzer (Foss A/S, Hillerød, Denmark).

On the same day as the milk sampling, blood samples (5 mL) from each cow were collected after the morning milking and before feeding by jugular venipuncture using vacutainer tubes containing 150 USP units of lithium heparin as anti-coagulant (Vacumed; FL Medical, Torreglia, Padua, Italy). All blood samples were kept on ice until centrifugation at 3500×*g* for 1 min at 6 °C (Hettich Universal 16R Centrifuge) within 2 h of blood sampling. The plasma obtained was collected and stored at − 20 °C until the blood metabolite assay was performed.

### FTIR spectra

Milk FTIR spectra were recorded and analyzed with a MilkoScan FT6000 (Foss A/S, Hillerød, Denmark), which covers transmittance values at 1060 wavenumbers ranging from 5011 to 925 (cm^−1^) from the short-wavelength infrared (SWIR) to the long-wavelength infrared (LWIR) regions. The whole-milk FTIR (n = 1060), without removing the water absorption regions, were used to develop prediction equations. Two milk spectra were obtained for each sample and were expressed as absorbance values [log(1/transmittance)], then standardized to a mean of 0 and a standard deviation of 1; the values were then averaged before data analysis. Milk FTIR quality control was carried out using principal component analysis and Mahalanobis distance at a significance level of 5% to remove possible outlier animals, i.e., those with large differences in their FTIR information, according to Shah and Gemperline [32]. After quality control, milk spectra from 1140 cows remained for further analysis. Among the 10 animals that were excluded from the analysis, none showed outlier values for blood metabolites.

### Genotyping

In total, 1067 Holstein cows were genotyped with the GGP (Geneseek Genomic Profiler) Bovine 100K single nucleotide polymorphism (SNP) Chip assay. After removing SNPs in the non-autosomal regions, quality control of the genotypes was carried out. Autosomal SNPs with a minor allele frequency (MAF) lower than 0.05, SNPs that showed a significant deviation from Hardy–Weinberg equilibrium (*P* ≤ 10^−5^), and SNPs and samples with a call rate lower than 0.95 were removed. After quality control, 1055 cows and 80,274 SNPs remained for further analyses.

### Blood metabolic profiling

Blood metabolic profiles were assessed for biomarkers that are associated with energy metabolism (glucose, BHBA and urea), inflammation/innate immune response (ceruloplasmin and total proteins [PROTt]), liver function/hepatic damage (AST, GGT and PON), oxidative stress metabolites (advanced oxidation protein products [AOPP], ferric reducing antioxidant power [FRAP] and total reactive oxygen metabolites [ROMt]) and minerals (calcium, potassium, and zinc).

We used the clinical auto-analyzer (ILAB 650, Instrumentation Laboratory, Lexington, MA) to determine the concentrations of glucose, PROTt, albumin, urea, calcium, AST, and GGT in the plasma samples using kits that were purchased from Instrumentation Laboratory (IL Test). Globulin concentration was estimated as the difference between total proteins and albumin. The potassium electrolyte (K^+^) was detected by the potentiometer method using an Ion Selective Electrode coupled to the ILAB 600 analyzer. Zinc, BHBA, and ceruloplasmin were analyzed using the methods described in Calamari et al. [33]. The concentration of ROMt, FRAP and PON were determined according to Premi et al. [34], and those of AOPP according to Hanasand et al*.* [35]. Missing information was excluded and after data integration, 1020 cows with data on blood metabolites, milk FTIR data and genomic information remained for further analyses. The descriptive statistics for the blood metabolites are in Table 1, a density plot for each blood metabolite is displayed in Additional file 2: Fig. S1, and the phenotypic means for the blood metabolites across lactation are in Additional file 1: Table S1 and Additional file 2: Fig. S2.

**Table 1:** Descriptive statistics for blood metabolites related to energy, liver function/hepatic damage, oxidative stress, inflammation/innate immunity, and minerals

Hemato-chemical parameters	N	Mean	SD	P1	P99
Energy-related metabolites					
Glucose, mmol/L	1019	4.35	0.33	3.54	5.12
BHBA, mmol/L	1012	0.53	0.17	0.26	1.12
Urea, mmol/L	1018	6.40	1.05	3.99	8.90
Liver function/hepatic damage					
AST, U/L	1013	100.65	21.72	62.93	169.59
GGT, U/L	1014	28.36	7.16	14.68	47.75
PON, U/mL	1018	97.28	19.29	55.54	147.67
Oxidative stress metabolites					
ROMt, mgH_2_O_2_/100 mL	1017	12.70	3.06	6.59	21.36
AOPP, µmol/L	1019	48.05	9.14	27.20	73.70
FRAP, µmol/L	1014	195.96	36.62	120.96	286.26
Inflammation/innate immunity					
Ceruloplasmin, µmol/L	1018	1.90	0.60	0.75	3.63
PROTt, g/L	1017	81.25	4.82	72.03	95.60
Globulins, g/L	1013	43.76	5.36	34.88	60.91
Minerals					
Calcium, mmol/L	1018	2.51	0.11	2.21	2.77
Potassium, mmol/L	1016	4.13	0.41	3.17	5.20
Zinc, µmol/L	1012	12.02	2.64	6.36	20.25

BHBA: β-hydroxybutyric acid; AST: aspartate aminotransferase; GGT: γ-glutamyl transferase; PON: paraoxonase; ROMt: total reactive oxygen metabolites; AOPP: advanced oxidation protein products; FRAP: ferric reducing antioxidant power; PROTt: total proteins; N: number of records; SD: standard deviation; P1: 1st percentile; P99: 99th percentile

### Genetic parameters

We inferred the genetic parameters for the blood metabolites using an animal model via a single-step genomic best linear unbiased prediction (ssGBLUP) model, as follows:

$$\mathbf{y}=\mathbf{X}\mathbf{b}+\mathbf{W}\mathbf{h}+\mathbf{Z}\mathbf{a}+\mathbf{e},$$

where $\mathbf{y}$ is the vector of the phenotypic data for blood metabolites; $\mathbf{b}$ is the vector of fixed effects for DIM (6 classes, i.e. class 1: less than 60 days; class 2: 60–120 days; class 3: 121–180 days; class 4: 181–240 days; class 5: 241–300 days; and class 6: > 300 days), and parity (four classes: 1, 2, 3, ≥ 4); $\mathbf{h}$ is the vector of the random effect of batch; $\mathbf{a}$ is the vector of additive genetic effects; $\mathbf{X}$, $\mathbf{W}$, and $\mathbf{Z}$ are the incidence matrices relating $\mathbf{y}$ to the fixed effects ($\mathbf{b}$), the batch effect ($\mathbf{h}$), and the additive genetic effects ($\mathbf{a}$), respectively; and $\mathbf{e}$ is the vector of random residual effects.

The ssGBLUP model was fitted under the following assumptions for the random effects: ${\mathbf{a}}\sim N\left( {{\mathbf{0}},{\mathbf{H}}{\mathbf{ \otimes \sigma }}_{{\text{a}}}^{2} } \right)$, ${\mathbf{h}}\sim N\left( {{\mathbf{0}},{\mathbf{I}}{\mathbf{ \otimes }}{\upsigma }_{{{\text{batch}}}}^{2} } \right)$, and ${\mathbf{e}}\sim N\left( {{\mathbf{0}},{\mathbf{I}}{\mathbf{ \otimes \sigma }}_{{\text{e}}}^{2} } \right)$, where $\sigma_{{\text{a}}}^{2} ,\;\sigma_{{{\text{batch}}}}^{2} ,\sigma_{{\text{e}}}^{2}$ are the variances for the additive, batch, and residual effects, respectively, $\mathbf{I}$ is the identity matrix, and the symbol ${\mathbf{ \otimes }}$ represents the Kronecker product. $\mathbf{H}$ is a matrix that combines pedigree and genomic information [36], and its inverse $\left({\mathbf{H}}^{\mathbf{-1}}\right)$ is given by: $${\mathbf{H}}^{\mathbf{-1}}={\mathbf{A}}^{\mathbf{-1}}+\left[\begin{array}{cc}{\mathbf{0}}& {\mathbf{0}}\\ {\mathbf{0}}& {\mathbf{G}}^{\mathbf{-1}} - {\mathbf{A}}_{\mathbf{22}}^{\mathbf{-1}}\end{array}\right]$$, where ${\mathbf{A}}^{\mathbf{-1}}$ is the inverse of the pedigree relationship matrix, ${\mathbf{A}}_{\mathbf{22}}^{\mathbf{-1}}$ is the inverse of the pedigree relationship matrix for the genotyped animals, and ${\mathbf{G}}^{\mathbf{-1}}$ is the inverse of the genomic relationship matrix obtained according to VanRaden [37]. The pedigree relationship matrix was built from pedigree information considering three generations. The $\mathbf{G}$ matrix was built as follows: $\mathbf{G}=\frac{\mathbf{M}{\mathbf{M}}^{\mathbf{^{\prime}}}}{2\sum_{j=1}^{m}{p}_{j}\left(1-{p}_{j}\right)}$ where $\mathbf{M}$ is the SNP matrix assuming 0, 1, and 2 for genotypes *AA*, *AB*, and *BB*; and ${p}_{j}$ is the frequency of the second allele at the $j$th SNP.

We assumed a flat prior distribution for the fixed effects and used an inverse Wishart distribution as a prior for the random effects. The heritability (${h}^{2}$) was calculated based on the posterior variance estimates for each trait as ${h}^{2}={\upsigma }_{\mathrm{a}}^{2}/({\upsigma }_{\mathrm{a}}^{2}+{\upsigma }_{\mathrm{e}}^{2})$, and the batch incidence (${h}_{\mathrm{batch}}$) was estimated as ${h}_{\mathrm{batch}}={\upsigma }_{\mathrm{batch}}^{2}/({\upsigma }_{\mathrm{a}}^{2}+{\upsigma }_{\mathrm{batch}}^{2}+{\upsigma }_{\mathrm{e}}^{2})$.

The model was implemented in the gibbsf90 + software from the blupf90 family of programs [38]. The estimates of genetic parameters were sampled from the posterior distribution using the Gibbs sampling algorithm. A single chain consisting of 500,000 cycles was used, with a burn-in of the first 100,000 iterations and with samples stored every five cycles. Hence, the posterior means were obtained from 80,000 samples. The analysis converged through visual inspection using the boa package in R [39], and converged with a p-value > 0.05 for the Geweke test [40].

### Cross-validation scenarios

The predictive ability (${\mathrm{R}}^{2}$) for each blood metabolite using the gradient boosting machine (GBM) and the BayesB approaches was assessed with two random cross-validation (CV) scenarios (tenfold, and batch-out) and an independent CV scenario (herd-out). The training and validation sets in the tenfold CV and batch-out CV scenarios were fixed for both models. In the tenfold CV*,* the dataset was randomly split into ten non-overlapping folds of approximately equal size (100 to 102 cows per fold). Nine folds were assigned to the training set and one to the validation set. To evaluate the reliability of the model, the CV scenario was repeated ten times, such that each fold was predicted in the validation set once.

In the batch-out CV scenario, the training and validation sets were assigned according to the Euclidean distance of the genomic relationship of animals across the batches (n = 16), with the aim to create independence between training and validation sets. The training set consisted of 80% of the batches (n = 13), while the other 20% (n = 3) were assigned to the validation set, with both sets being independent of each other. The assessment of the predictive ability in this CV scenario was repeated five times to ensure that each batch was assigned once to the validation set to be predicted. The final R^2^ for both CV scenarios were estimated for each replicate, and their average was taken as the predictive ability, while the SD was taken as the variability in predictive ability. For the herd-out CV scenario, two combinations were assessed i.e., one that considered herd 1 (n = 945) as the training set and herd 2 (n = 75) as the validation set and one that considered herd 2 as the training set and herd 1 as the validation set.

### Statistical analyses

The target blood metabolite traits were predicted using four statistical models with increasing complexity: Model 1 (M1), the baseline model, which included only milk FTIR data; Model 2 (M2), which included milk FTIR data and on-farm information (DIM and parity); Model 3 (M3), which included milk FTIR data and genomic information; and Model 4 (M4), which included milk FTIR data, on-farm and genomic information. These four models were used to evaluate the predictive ability of the GBM [41] and BayesB [42] algorithms. The water absorbance region in the FTIR spectra was not removed because the statistical approaches used, i.e., GBM and BayesB can handle situations where noise is present in the predictor variables.

GBM is an ensemble learning algorithm that uses boosting to convert weak regression tree models into strong learners [41, 43] by combining different predictor variables sequentially in the regression tree model, selecting and shrinking the predictor variables to control the residual from the previous model [44]. To obtain the best results from the GBM algorithm, a random search was used to determine the best combination of four major hyperparameters that maximized the predictive ability for each trait. The GBM algorithm was implemented using the *h2o* R package (https://cran.r-project.org/web/packages/h2o). For the random search, we considered the number of trees (ntree) ranging from 100 to 3000 in intervals of 20, the learning rate (lrn_rate) with values from 0 to 1 in intervals of 0.1, maximum tree depth (max_depth) ranging from 5 to 80 in intervals of 5, and the minimum samples per leaf ranging from 20 to 100 in intervals of 20. The random grid search was performed in the *h2o.grid* function of the *h2o* R package (https://cran.r-project.org/web/packages/h2o) with a maximum of 100 models with random combinations of the hyperparameters. For this, the training set for each CV scenario was split into 5 folds during the learning process of the GBM approach and the trained model with the highest predictive ability (${\mathrm{R}}^{2}$) and the lowest mean square error (MSE) was applied to the disjoint validation set of each CV scenario. The relative importance of the FTIR wavelength information (i.e., variable importance [VI]) was determined by calculating the relative influence of the improvements in predictive ability during the tree regression building process, which was the sum of the squared improvements over all the internal nodes of the tree for which the FTIR wavelength was chosen as the partitioning variable [41].

The BayesB analyses were performed using the implementation in the R package BGLR version 1.09 [42]. The BGLR default priors were used for all models, with 5 degrees of freedom (${\mathrm{df}}_{\mathrm{u}}$), a scale parameter (${\mathrm{S}}_{\mathrm{x}}$), and $\uppi$. BayesB assumes that most independent variables (${w}_{eff}$) have a null effect (i.e., a point mass at 0), and that a few independent variables contribute to explaining the variability of the target trait [45, 46]. The prior conditional distribution on the independent variable is assumed to be a mixture with a point mass at 0 $\left[\left(1-\uppi \right){*(w}_{eff}=0\right)]$ i.e., the probability of SNPs with null effects and a scaled t distribution $[\uppi *\mathrm{t}\left({w}_{eff}|\mathrm{df},{\mathrm{S}}_{\mathrm{B}}\right)]$ as follows:

$$\mathrm{p}\left({w}_{eff} |\mathrm{df},\uppi ,{\mathrm{S}}_{\mathrm{B}}\right)=\uppi *\mathrm{t}\left({w}_{eff}|\mathrm{df},{\mathrm{S}}_{\mathrm{B}}\right)+\left(1-\uppi \right)*\left({w}_{eff}=0\right),$$

where $$\uppi$$ represents the proportion of the non-null effect, and $$1-\uppi$$ is the proportion of the null effect of the independent variable that contributes to the variability of the target trait [46]; $${w}_{eff}$$ is the effect of the independent variable, $$\mathrm{t}\left({w}_{eff}|\mathrm{df},{\mathrm{S}}_{\mathrm{B}}\right)$$ is a scaled t distribution with df = 5 degrees of freedom and $${\mathrm{S}}_{\mathrm{B}}$$ scale $$({\mathrm{S}}_{\mathrm{B}}=\mathrm{var}\left(\mathrm{y}\right)*VP*(\mathrm{df}+2)/{\mathrm{MS}}_{\mathrm{x}}/\uppi$$), were $${\mathrm{MS}}_{\mathrm{x}}$$ represents the sum of the variances of the columns of X, and $$VP=0.5$$ is the variance partition used to control the variance attributable to the linear predictors, in this case 50% by program default. The residual variance was assigned a scaled-inverse $${\upchi }^{2}$$ density with degrees of freedom, $${\mathrm{df}}_{\mathrm{e}}$$, and scale, $${\mathrm{S}}_{\mathrm{e}}$$, ($${\mathrm{S}}_{\mathrm{e}}=\mathrm{var}\left(\mathrm{y}\right)*{\mathrm{R}}^{2}*({\mathrm{df}}_{\mathrm{e}}+2)$$). For the SNP effects, we used the default prior parameters $$\uppi =0.5$$ and $$count=10$$ to assign the beta-prior for two fixed shape parameters and derived the proportion of nonzero SNP effects $$\uppi$$ for the SNP term [42]. Thus, the two shape parameters of the beta distribution $${\uppi }_{0}=\frac{0.5*(1-0.5)}{10+1}=0.023$$ and $${p}_{0}=0.5$$ [29].

The predictive ability of the BayesB and GBM approaches for the four models (M1, M2, M3 and M4) on the validation set was assessed by the coefficient of determination (${\mathrm{R}}^{2}$) between the observed and predicted phenotypes. In addition, the standard deviations (SD) of the ${\mathrm{R}}^{2}$ values were also calculated across the ten replicates used in the CV scenarios. The second parameter used to assess model performance was the root mean squared error (RMSE) defined as: $\sqrt{\frac{\sum_{\mathrm{i}=1}^{\mathrm{n}}({\mathrm{y}}_{\mathrm{i}}-{\widehat{\mathrm{y}}}_{\mathrm{i}}{)}^{2}}{\mathrm{n}}}$ where $\mathrm{n}$ is the number of cows in the validation set. The linear regression slope between the observed (${\mathrm{y}}_{\mathrm{i}}$) and predicted values (${\widehat{\mathrm{y}}}_{\mathrm{i}}$) for each model and each cross-validation scenario was used to assess the degree of bias of the model. The relative difference (RD) in predictive ability is measured as $\mathrm{RD}=\frac{({\mathrm{r}}_{\mathrm{m}}-{\mathrm{r}}_{\mathrm{M}1})}{{\mathrm{r}}_{\mathrm{M}1}}\times 100$, where ${\mathrm{r}}_{\mathrm{m}}$ is the predictive ability using Models M2, M3 or M4, and ${\mathrm{r}}_{\mathrm{M}1}$ is the predictive ability using the baseline Model M1.

## Results

### Variance components and heritability estimates

Posterior means of the variance components, heritability (${h}^{2}$) and batch incidence (${h}_{batch}$) for the blood metabolites are in Table 2. The lowest heritability estimates were observed for BHBA (0.12 ± 0.049), urea (0.18 ± 0.068), oxidative stress metabolite-related traits (ROMt—0.13 ± 0.06; AOPP—0.09 ± 0.045, FRAP—0.05 ± 0.037), total proteins (0.09 ± 0.044), globulins (0.09 ± 0.05) and calcium (0.12 ± 0.049). Moderate heritability estimates were observed for glucose (0.36 ± 0.083), AST (0.36 ± 0.091), GGT (0.43 ± 0.073), ceruloplasmin (0.37 ± 0.091), potassium (0.24 ± 0.067), and zinc (0.35 ± 0.078). A high heritability estimate was obtained for PON (0.60 ± 0.086). Batch incidences, calculated as the ratio between batch variance and total phenotypic variance, had relatively low values for liver function/hepatic damage traits, inflammation/innate immunity metabolite groups and ROMt; moderate values for BHBA, potassium, calcium, AOPP, and FRAP (ranging from 0.16 for BHBA to 0.28 for potassium), but showed a substantial contribution to the observed variability in glucose (0.44 ± 0.097), zinc (0.41 ± 0.092), and urea (0.36 ± 0.091).

**Table 2:** Estimates (± SD) of genetic (${\sigma}_{a}^{2}$), batch (${{{\sigma}}}_{{{b}}{{a}}{{t}}{{c}}{{h}}}^{2}$) and residual (${{{\sigma}}}_{{{e}}}^{2}$) variance components, heritability (${{{h}}}^{2}$), and batch incidence (${{{h}}}_{{{b}}{{a}}{{t}}{{c}}{{h}}}$) for blood metabolites

Traits	${\upsigma }_{\mathrm{a}}^{2}$	${\upsigma }_{\mathrm{batch}}^{2}$	${\upsigma }_{\mathrm{e}}^{2}$	${h}^{2}$	${h}_{\mathrm{batch}}$
Energy-related metabolites					
Glucose, mmol/L	0.018 ± 0.009	0.040 ± 0.035	0.032 ± 0.007	0.360 ± 0.083	0.444 ± 0.097
BHBA, mmol/L	0.002 ± 0.002	0.003 ± 0.003	0.014 ± 0.002	0.125 ± 0.049	0.158 ± 0.051
Urea, mmol/L	0.097 ± 0.061	0.305 ± 0.221	0.443 ± 0.061	0.180 ± 0.068	0.361 ± 0.091
Liver function/hepatic damage					
AST, U/L	136.228 ± 39.725	46.593 ± 18.297	241.761 ± 30.869	0.360 ± 0.091	0.110 ± 0.060
GGT, U/L	17.049 ± 4.153	0.528 ± 0.288	22.922 ± 3.945	0.427 ± 0.073	0.013 ± 0.013
PON, U/mL	197.144 ± 34.858	26.971 ± 13.834	129.064 ± 25.22	0.604 ± 0.086	0.076 ± 0.035
Oxidative stress metabolites					
ROMt, mgH_2_O_2_/100 mL	0.972 ± 0.485	0.677 ± 0.268	6.481 ± 0.915	0.130 ± 0.065	0.083 ± 0.040
AOPP, µmol/L	5.374 ± 2.769	19.491 ± 8.748	54.696 ± 3.459	0.089 ± 0.045	0.245 ± 0.073
FRAP, µmol/L	45.411 ± 4.575	295.345 ± 114.219	867.563 ± 155.12	0.050 ± 0.027	0.244 ± 0.074
Inflammation/innate immunity					
Ceruloplasmin, µmol/L	0.111 ± 0.034	0.023 ± 0.014	0.186 ± 0.028	0.374 ± 0.091	0.072 ± 0.034
PROTt, g/L	1.785 ± 0.786	1.401 ± 0.883	17.964 ± 1.254	0.090 ± 0.044	0.066 ± 0.034
Globulins, g/L	2.155 ± 0.955	1.636 ± 0.809	21.460 ± 2.059	0.091 ± 0.047	0.065 ± 0.033
Minerals					
Calcium, mmol/L	0.001 ± 0.001	0.002 ± 0.001	0.007 ± 0.001	0.125 ± 0.049	0.200 ± 0.073
Potassium, mmol/L	0.026 ± 0.007	0.042 ± 0.018	0.079 ± 0.009	0.248 ± 0.057	0.286 ± 0.080
Zinc, µmol/L	1.168 ± 0.444	2.377 ± 1.556	2.205 ± 0.377	0.346 ± 0.078	0.413 ± 0.092

BHBA: β-hydroxybutyric acid; AST: aspartate aminotransferase; GGT: γ-glutamyl transferase; PON: paraoxonase; ROMt: total reactive oxygen metabolites; AOPP: advanced oxidation protein products; FRAP: ferric reducing antioxidant power; PROTt: total proteins

$${\mathrm{h}}^{2}=\frac{{\upsigma }_{\mathrm{a}}^{2}}{{\upsigma }_{\mathrm{a}}^{2}+{\upsigma }_{\mathrm{e}}^{2}}$$

$${\mathrm{h}}_{\mathrm{batch}}={\upsigma }_{\mathrm{batch}}^{2}/({\upsigma }_{\mathrm{a}}^{2}+{\upsigma }_{\mathrm{batch}}^{2}+{\upsigma }_{\mathrm{e}}^{2})$$

### Predictive performance of FTIR data integrated with on-farm and genomic information

Model M1 (which included only the milk FTIR data) achieved the lowest R^2^ for each CV scenario and approach, compared with the models including on-farm data (M2), genomic information (M3) and both on-farm and genomic information (M4). For M1, the ${\mathrm{R}}^{2}$ values ranged from 0.45 to 0.85 for the tenfold CV, from 0.38 to 0.81 for the batch-out CV and from 0.23 to 0.74 for the herd-out CV (see Additional file 1: Tables S2, S3 and S4, respectively); for M2, they ranged from 0.47 to 0.88 for the tenfold CV, from 0.39 to 0.82 for the batch-out CV and from 0.24 to 0.75 for the herd-out CV (see Additional file 1: Tables S2, S3 and S4, respectively); for M3, they ranged from 0.49 to 0.89 for the tenfold CV, from 0.40 to 0.84 for the batch-out CV and from 0.25 to 0.78 for the herd-out CV (see Additional file 1: Tables S2, S3 and S4, respectively); and for M4, they ranged from 0.53 to 0.92 for the tenfold CV (Table 3), from 0.41 to 0.88 for the batch-out CV (Table 4) and from 0.25 to 0.80 for the herd-out CV (Table 5).

**Table 3:** Average milk Fourier transform infrared (FTIR) prediction performance (± SD) considering the systematic effect of days in milk, parity, and genomic information (Model M4) for the tenfold random cross-validation scenario of blood metabolites parameters using BayesB and gradient boosting machine (GBM) methods

Trait	Method					
	BayesB			GBM		
	R^2^	RMSE	Slope	R^2^	RMSE	Slope
Energy-related metabolites						
Glucose, mmol/L	0.80 ± 0.035	0.16 ± 0.008	1.03 ± 0.074	0.85 ± 0.029	0.14 ± 0.008	1.02 ± 0.044
BHBA, mmol/L	0.59 ± 0.059	0.11 ± 0.009	1.04 ± 0.097	0.62 ± 0.053	0.10 ± 0.006	0.99 ± 0.043
Urea, mmol/L	0.76 ± 0.041	0.52 ± 0.041	1.03 ± 0.099	0.80 ± 0.033	0.48 ± 0.042	1.01 ± 0.082
Liver function/hepatic damage						
AST, U/L	0.53 ± 0.085	13.53 ± 1.274	1.04 ± 0.174	0.63 ± 0.05	12.71 ± 0.539	1.01 ± 0.082
GGT, U/L	0.63 ± 0.063	4.12 ± 0.511	1.04 ± 0.130	0.65 ± 0.073	3.95 ± 0.350	1.01 ± 0.072
PON, U/mL	0.66 ± 0.031	11.59 ± 0.976	1.02 ± 0.105	0.69 ± 0.025	11.05 ± 0.471	0.99 ± 0.083
Oxidative stress metabolites						
ROMt, mgH_2_O_2_/100 mL	0.79 ± 0.059	1.51 ± 0.326	1.04 ± 0.095	0.82 ± 0.034	1.30 ± 0.090	1.01 ± 0.052
AOPP, µmol/L	0.68 ± 0.052	5.36 ± 0.439	1.03 ± 0.136	0.71 ± 0.043	4.92 ± 0.340	0.99 ± 0.092
FRAP, µmol/L	0.53 ± 0.053	24.86 ± 1.524	1.05 ± 0.173	0.58 ± 0.049	20.57 ± 1.158	0.98 ± 0.098
Inflammation/innate immunity						
Ceruloplasmin, µmol/L	0.77 ± 0.048	0.32 ± 0.049	1.02 ± 0.089	0.82 ± 0.027	0.28 ± 0.018	0.99 ± 0.073
PROTt, g/L	0.85 ± 0.029	1.90 ± 0.191	1.03 ± 0.079	0.88 ± 0.024	1.58 ± 0.106	1.02 ± 0.052
Globulins, g/L	0.89 ± 0.019	1.82 ± 0.186	1.02 ± 0.078	0.92 ± 0.023	1.68 ± 0.133	1.01 ± 0.033
Minerals						
Calcium, mmol/L	0.61 ± 0.063	0.07 ± 0.006	1.05 ± 0.132	0.67 ± 0.032	0.05 ± 0.005	1.03 ± 0.098
Potassium, mmol/L	0.67 ± 0.055	0.24 ± 0.015	1.03 ± 0.102	0.72 ± 0.027	0.21 ± 0.015	1.02 ± 0.082
Zinc, µmol/L	0.63 ± 0.032	1.54 ± 0.054	1.04 ± 0.222	0.70 ± 0.033	1.44 ± 0.057	0.98 ± 0.095

BHBA = β-hydroxybutyric acid; AST = aspartate aminotransferase; GGT : γ-glutamyl transferase; PON = paraoxonase; ROMt = total reactive oxygen metabolites; AOPP = advanced oxidation protein products; FRAP = ferric reducing antioxidant power; PROTt = total proteins, RMSE: root mean squared error

R^2^: coefficient of determination between the observed and predicted phenotypes in validation set and standard deviation (SD) as the variability measurement of predictive ability

**Table 4:** Average milk Fourier transform infrared (FTIR) prediction performance (± SD) considering the systematic effect of days in milk, parity, and genomic information (Model M4) for the batch-out cross-validation scenario of blood metabolites parameters using BayesB and gradient boosting machine (GBM) methods

Trait	Method					
	BayesB			GBM		
	R^2^	RMSE	Slope	R^2^	RMSE	Slope
Energy-related metabolites						
Glucose, mmol/L	0.74 ± 0.054	0.17 ± 0.012	1.04 ± 0.073	0.79 ± 0.051	0.16 ± 0.015	0.99 ± 0.060
BHBA, mmol/L	0.53 ± 0.068	0.12 ± 0.034	1.05 ± 0.117	0.58 ± 0.066	0.11 ± 0.004	0.98 ± 0.078
Urea, mmol/L	0.64 ± 0.050	0.54 ± 0.082	0.96 ± 0.093	0.74 ± 0.055	0.49 ± 0.048	1.01 ± 0.075
Liver function/hepatic damage						
AST, U/L	0.51 ± 0.093	15.34 ± 1.415	1.04 ± 0.136	0.61 ± 0.053	14.31 ± 0.828	1.01 ± 0.087
GGT, U/L	0.53 ± 0.034	5.06 ± 0.341	1.05 ± 0.056	0.60 ± 0.043	4.95 ± 0.259	1.02 ± 0.090
PON, U/mL	0.59 ± 0.045	12.46 ± 1.149	0.97 ± 0.095	0.64 ± 0.049	12.13 ± 0.658	1.01 ± 0.083
Oxidative stress metabolites						
ROMt, mgH_2_O_2_/100 mL	0.74 ± 0.040	1.58 ± 0.149	1.03 ± 0.050	0.77 ± 0.022	1.41 ± 0.152	0.99 ± 0.065
AOPP, µmol/L	0.54 ± 0.042	5.68 ± 0.423	1.07 ± 0.073	0.63 ± 0.072	5.5 ± 0.449	1.02 ± 0.074
FRAP, µmol/L	0.41 ± 0.084	23.58 ± 3.552	1.09 ± 0.077	0.48 ± 0.065	21.5 ± 1.572	1.01 ± 0.076
Inflammation/innate immunity						
Ceruloplasmin, µmol/L	0.68 ± 0.066	0.33 ± 0.033	0.95 ± 0.124	0.74 ± 0.056	0.31 ± 0.025	0.99 ± 0.069
PROTt, g/L	0.77 ± 0.027	2.09 ± 0.172	0.96 ± 0.042	0.85 ± 0.040	1.87 ± 0.183	1.01 ± 0.045
Globulins, g/L	0.82 ± 0.035	2.23 ± 0.230	1.07 ± 0.037	0.88 ± 0.025	1.96 ± 0.150	1.03 ± 0.029
Minerals						
Calcium, mmol/L	0.53 ± 0.030	0.07 ± 0.006	0.94 ± 0.099	0.62 ± 0.096	0.06 ± 0.023	1.01 ± 0.046
Potassium, mmol/L	0.58 ± 0.069	0.23 ± 0.015	1.05 ± 0.093	0.67 ± 0.078	0.22 ± 0.014	1.01 ± 0.078
Zinc, µmol/L	0.57 ± 0.054	1.63 ± 0.341	1.06 ± 0.085	0.67 ± 0.071	1.46 ± 0.244	1.03 ± 0.067

BHBA: β-hydroxybutyric acid; AST: aspartate aminotransferase; GGT: γ-glutamyl transferase; PON: paraoxonase; ROMt: total reactive oxygen metabolites; AOPP: advanced oxidation protein products; FRAP: ferric reducing antioxidant power; PROTt: total proteins; RMSE: root mean squared error

R^2^: coefficient of determination between the observed and predicted phenotypes in validation set and standard deviation (SD) as the variability measurement of predictive ability

**Table 5:** Average milk Fourier transform infrared (FTIR) prediction performance (± SD) considering the systematic effect of days in milk, parity, and genomic information (Model M4) for the herd-out cross-validation scenario of blood metabolites parameters using BayesB and gradient boosting machine (GBM) methods

Trait	Method					
	BayesB			GBM		
	R^2^	RMSE	Slope	R^2^	RMSE	Slope
Energy-related metabolites						
Glucose, mmol/L	0.63 ± 0.066	0.26 ± 0.034	1.07 ± 0.040	0.65 ± 0.035	0.16 ± 0.008	1.02 ± 0.073
BHBA, mmol/L	0.40 ± 0.105	0.21 ± 0.033	0.94 ± 0.068	0.43 ± 0.062	0.12 ± 0.009	0.97 ± 0.057
Urea, mmol/L	0.47 ± 0.164	0.78 ± 0.119	0.97 ± 0.084	0.49 ± 0.051	0.52 ± 0.041	1.01 ± 0.093
Liver function/hepatic damage						
AST, U/L	0.38 ± 0.115	17.14 ± 2.069	0.97 ± 0.072	0.43 ± 0.085	13.53 ± 1.268	1.05 ± 0.060
GGT, U/L	0.45 ± 0.080	5.38 ± 0.341	0.98 ± 0.051	0.48 ± 0.063	4.12 ± 0.511	1.03 ± 0.046
PON, U/mL	0.48 ± 0.035	17.21 ± 0.127	0.99 ± 0.091	0.52 ± 0.081	12.59 ± 1.577	1.02 ± 0.062
Oxidative stress metabolites						
ROMt, mgH_2_O_2_/100 mL	0.66 ± 0.071	1.89 ± 0.133	0.97 ± 0.089	0.69 ± 0.079	1.51 ± 0.326	1.01 ± 0.056
AOPP, µmol/L	0.37 ± 0.057	8.86 ± 0.770	0.98 ± 0.076	0.41 ± 0.052	5.36 ± 0.440	1.01 ± 0.073
FRAP, µmol/L	0.25 ± 0.061	36.86 ± 1.664	0.94 ± 0.082	0.29 ± 0.093	25.85 ± 2.601	1.01 ± 0.097
Inflammation/innate immunity						
Ceruloplasmin, µmol/L	0.58 ± 0.052	0.44 ± 0.004	0.96 ± 0.058	0.62 ± 0.066	0.32 ± 0.050	1.01 ± 0.065
PROTt, g/L	0.76 ± 0.02	2.57 ± 0.255	0.97 ± 0.021	0.79 ± 0.039	1.91 ± 0.189	0.98 ± 0.050
Globulins, g/L	0.77 ± 0.011	2.6 ± 0.312	0.96 ± 0.047	0.80 ± 0.019	1.82 ± 0.186	0.97 ± 0.037
Minerals						
Calcium, mmol/L	0.42 ± 0.086	0.17 ± 0.004	1.01 ± 0.093	0.47 ± 0.073	0.07 ± 0.007	0.98 ± 0.099
Potassium, mmol/L	0.54 ± 0.065	0.32 ± 0.014	0.98 ± 0.094	0.57 ± 0.055	0.24 ± 0.014	1.04 ± 0.093
Zinc, µmol/L	0.47 ± 0.083	2.27 ± 0.056	1.05 ± 0.099	0.50 ± 0.042	1.54 ± 0.054	1.02 ± 0.095

BHBA: β-hydroxybutyric acid; AST: aspartate aminotransferase; GGT: γ-glutamyl transferase; PON: paraoxonase; ROMt: total reactive oxygen metabolites; AOPP: advanced oxidation protein products; FRAP: ferric reducing antioxidant power; PROTt: total proteins, RMSE: root mean squared error

R^2^: coefficient of determination between the observed and predicted phenotypes in validation set and standard deviation (SD) as the variability measurement of predictive ability

**Fig. 1:**
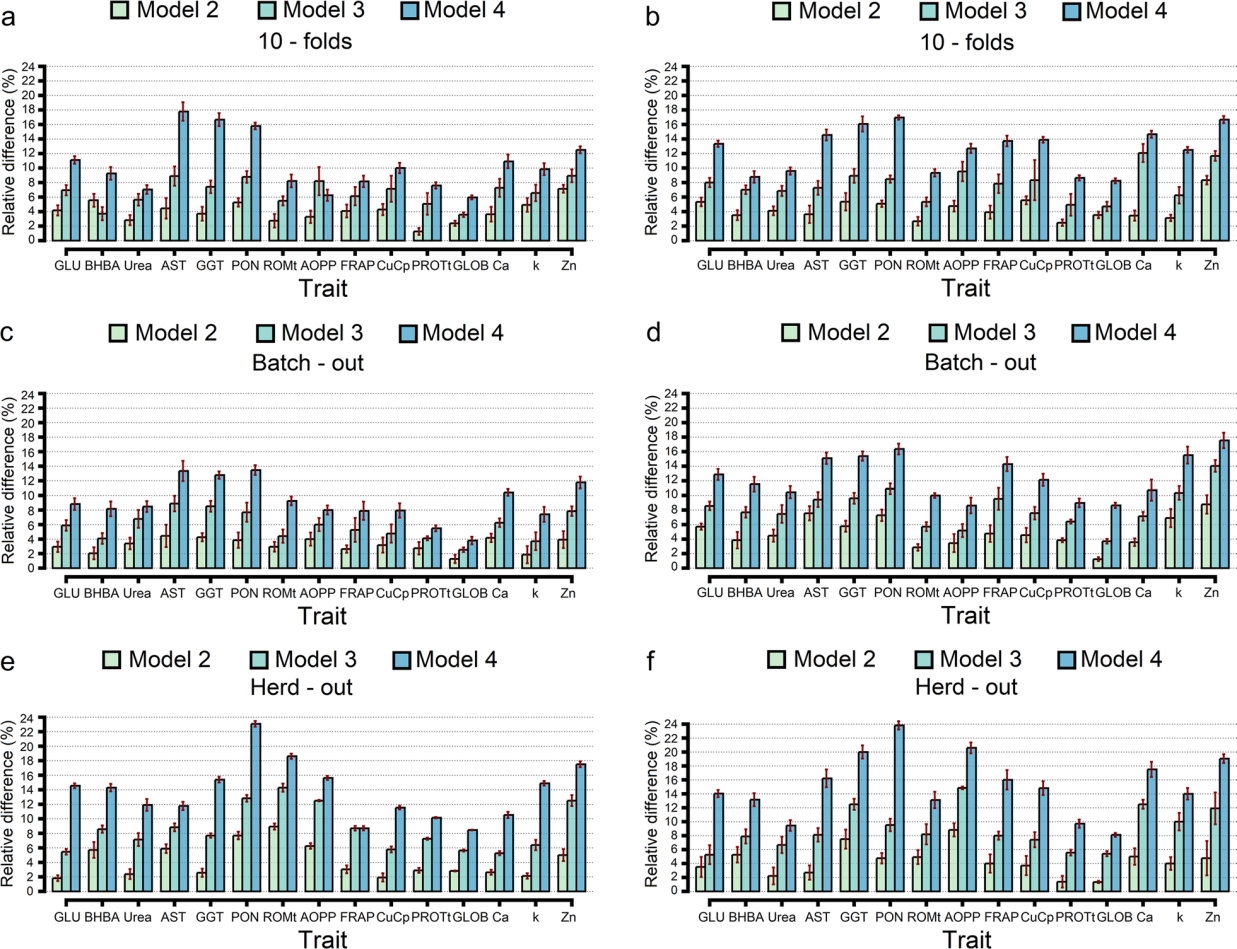
Average of the relative difference (%) in predictive ability across tenfold random, batch-out and herd-out cross-validation scenarios for Model 2 (milk FTIR data and on-farm data), Model 3 (milk FTIR data and genomic information) and Model 4 (milk FTIR data, on-farm data, and genomic information) for BayesB (**a**, **c**, **e**) and gradient boosting machine (GBM) (**b**, **d**, **f**) against the Model 1 considering only the FTIR data. Data are shown as mean ± SD (dark red error bar line) [for more details, see Additional file 1: Table S2 to S4]. GLU: Glucose; BHBA: β-hydroxybutyric acid; AST: aspartate aminotransferase; GGT: γ-glutamyl transferase; PON: paraoxonase; ROMt: total reactive oxygen metabolites; AOPP: advanced oxidation protein products; FRAP: ferric reducing antioxidant power; CuCp: Ceruloplasmin; PROTt: total proteins; GLOB: Globulin; Ca: Calcium; k: Potassium; Zn: Zinc

The relative differences (RD) in R^2^ obtained by the BayesB and GBM approaches with the tenfold CV, batch-out CV and herd-out CV showed great improvements in ${\mathrm{R}}^{2}$ for Models M2, M3 and M4 compared to Model M1 (Fig. 1). Integrating on-farm data in the baseline model (Model M2) increased the ${\mathrm{R}}^{2}$ by 1.27% to 7.14% using BayesB (Fig. 1a), and by 2.47% to 8.33% using GBM (Fig. 1b) with the tenfold CV. For the batch-out CV, RD ranged from 1.27 to 4.44% using BayesB (Fig. 1c) and from 1.23 to 8.77% using GBM (Fig. 1d), and for the herd-out CV they ranged from 1.82 to 8.93% using BayesB (Fig. 1e) and from 1.35 to 8.82% using GBM (Fig. 1f). When genomic information was integrated in the baseline model (Model M3), for the tenfold CV the observed RD ranged from 3.57 to 8.93% using BayesB (Fig. 1a) and from 4.71 to 12.07% using GBM (Fig. 1b), for the batch-out CV they ranged from 2.53 to 8.89% using BayesB (Fig. 1c) and from 3.70 to 14.04% using GBM (Fig. 1d), and for the herd-out CV they ranged from 5.45 to 14.29% using BayesB (Fig. 1e) and from 5.26 to 14.71% using GBM (Fig. 1f).

When both on-farm data and genomic information were integrated in the baseline model (Model 4), for the tenfold CV, RD ranged from 5.95 to 17.78% using BayesB (Fig. 1a) and from 8.24 to 16.95% using GBM (Fig. 1b); for the batch-out CV, they ranged from 3.80 to 13.46% using BayesB (Fig. 1c) and from 8.62 to 17.54% using GBM (Fig. 1d); and for the herd-out CV, they ranged from 8.45 to 23.08% using BayesB (Fig. 1e) and from 8.11 to 23.81% using GBM (Fig. 1f). Phenotypic predictions obtained with M4 were markedly improved, especially in the herd-out CV scenario for some of the blood metabolites such as PON (23.08%), ROMt (17.86%), AST (17.78%), and zinc (17.5%) using BayesB, and PON (23.81%), AOPP (20.59%), GGT (20%) zinc (19%) and calcium (17.5%) using GBM. In addition, RD values were higher than 11% for blood metabolites related to liver function/hepatic damage across all CV scenarios.

The slope coefficients obtained from all four models (M1, M2, M3 and M4) using BayesB or GBM showed that the predictions were slightly inflated (slope < 1) or deflated (slope > 1), with values ranging from 0.95 to 1.15 for the tenfold CV, from 0.90 to 1.17 for the batch-out CV and from 0.90 to 1.15 for the herd-out CV (Tables 3, 4, 5) and (see Additional file 1: Tables S2, S3 and S4). Overall, the slope values were lower with GBM than with BayesB across all evaluated CV scenarios.

### Comparing the statistical approaches across cross-validation scenarios

The predictive abilities of BayesB and GBM with Model M4 (integrating milk FTIR, DIM, parity, and SNP data) for blood metabolite-related traits ranged from moderate to high according to trait and model (Tables 3, 4, 5). Overall, the average predictions for inflammation/innate immunity traits (${\mathrm{R}}^{2}$ = 0.79, ranging from 0.58 to 0.92), and for energy-related metabolite traits (${\mathrm{R}}^{2}$ = 0.64, ranging from 0.40 to 0.85) were more accurate than the predictions for minerals (${\mathrm{R}}^{2}$ = 0.59, ranging from 0.42 to 0.72), oxidative stress metabolites (${\mathrm{R}}^{2}$ = 0.58, ranging from 0.25 to 0.82), and liver function/hepatic damage metabolites (${\mathrm{R}}^{2}$ = 0.56, ranging from 0.38 to 0.69) (see Tables 3, 4, 5).

The predictive ability of GBM was superior to that of BayesB (Tables 3, 4, 5), and the most remarkable differences in predictive ability between GBM and BayesB were observed with the tenfold CV for AST: 0.63 vs. 0.53 (18.9%), zinc: 0.70 vs. 0.63 (11.1%) and calcium: 0.67 vs. 0.61 (9.84%). With the batch-out CV, the largest differences in predictive ability between GBM and BayesB were observed for AST: 0.61 vs. 0.51 (19.6%), zinc: 0.67 vs. 0.57 (17.5%), FRAP: 0.48 vs. 0.41 (17.07%), calcium: 0.62 vs. 0.53 (16.98%), AOPP: 0.63 vs. 0.54 (16.67%), urea: 0.74 vs. 0.64 (15.63%), potassium: 0.67 vs. 0.58 (15.52%) and GGT: 0.60 vs. 0.53 (13.2%), and with the herd-out CV, the largest differences were observed for FRAP: 0.29 vs. 0.25 (16%), AST: 0.43 vs. 0.38 (13.16%), and calcium: 0.47 vs. 0.42 (11.9%). The slope coefficient was used to measure the extent of the prediction bias (Tables 3, 4, 5) and showed that the values for the predictions from GBM were closer to 1 than those from BayesB, which suggests that the predictions based on GBM are less biased.

### Effects of the heritability on the relative gains in predictive ability between Models 1 and 4 on blood metabolites

**Fig. 2:**
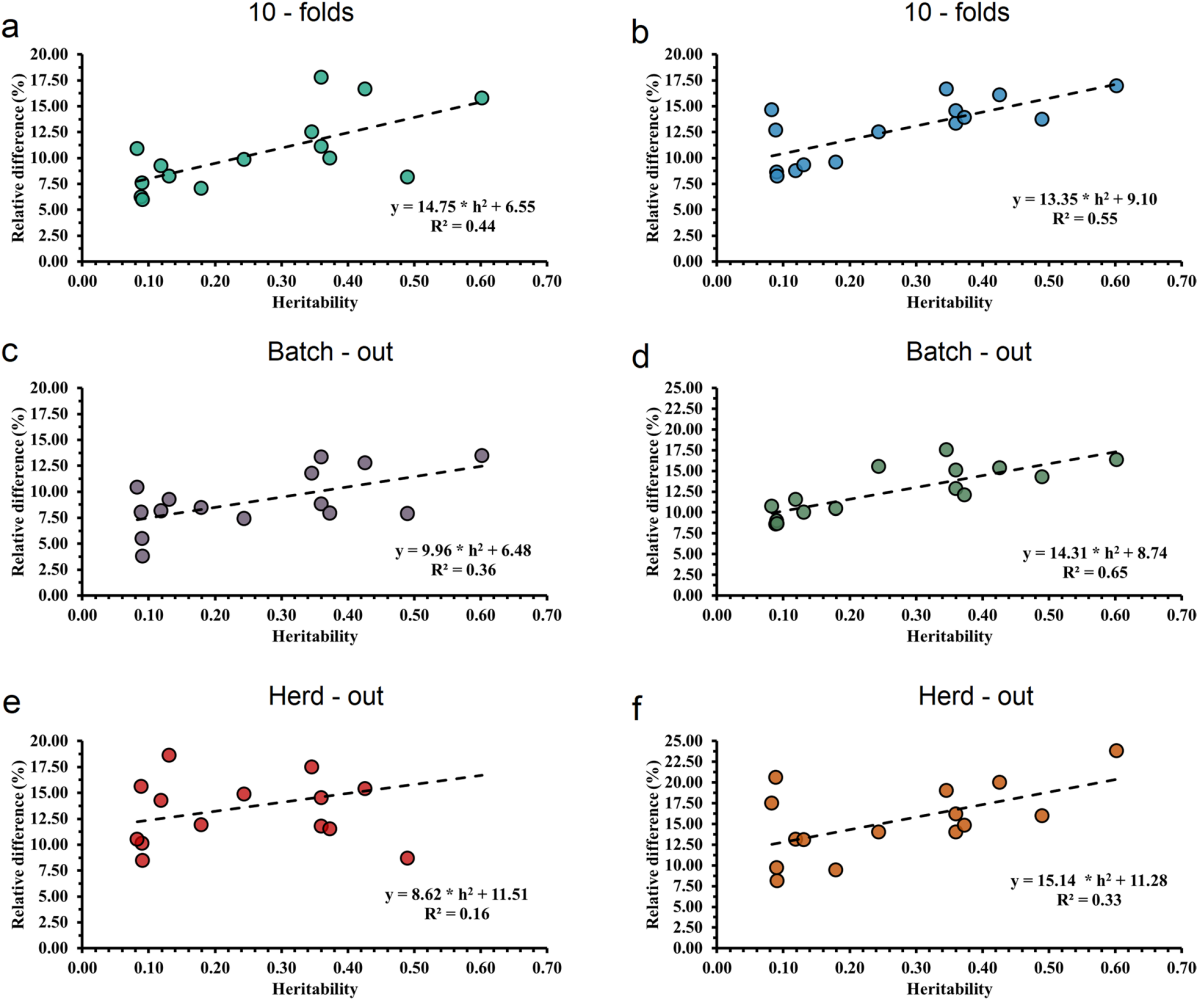
Relationship between relative difference (expressed as the difference in predictive ability from Model 4, i.e., full model including FTIR data, DIM, parity and genomic information and from Model 1 considering only FTIR data) and heritability estimates for each trait. Plots are differentiated for tenfold, batch-out and herd-out cross-validation scenarios using BayesB (**a**, **c**, **e**) and gradient boosting machine (**b**, **d**, **f**) approaches for predicted blood metabolites

The genetic architecture of blood metabolite traits is polygenic and controlled by several genes with moderate to large effects [47], which may affect phenotype prediction depending on the model used. The relative difference in predictive ability (${\mathrm{R}}^{2}$) for blood metabolites between Models M4 (full model) and M1 (base model, only milk FTIR) increased as the heritability estimate of the trait increased (Fig. 2). The ${\mathrm{R}}^{2}$ obtained from fitting a linear regression of the RD of M4 against M1 on the heritability estimates was used to evaluate the strength of their association. With the tenfold CV, ${\mathrm{R}}^{2}$ of 0.44 for BayesB (Fig. 2a) and of 0.55 for GBM (Fig. 2b), with the batch-out CV, ${\mathrm{R}}^{2}$ of 0.36 for BayesB (Fig. 2c) and of 0.65 for GBM (Fig. 2d) and with the herd-out CV ${\mathrm{R}}^{2}$ of 0.16 for BayesB (Fig. 2e) and of 0.33 for GBM (Fig. 2f) were obtained. As the heritability of the trait increased, the R^2^ increased with both statistical approaches and for all CV scenarios. The greatest gains in predictive ability were observed for PON (from 13.46 to 23.81%), GGT (from 12.77 to 20%), ceruloplasmin (from 7.94 to 14.81%), AST (from 11.76 to 17.78%), BHBA (from 8.16 to 14.29%), glucose (from 8.82 to 14.55%), and zinc (from 11.76 to 19.05%). Furthermore, considerable improvements in predictive ability were also observed for blood metabolites with lower heritability estimates, such as calcium (from 10.42 to 17.50%) and FRAP (from 7.89 to 16%). As expected, with both CV scenarios, GBM produced greater gains in predictive ability and stronger associations between its predictive ability and the heritability of blood metabolites (Fig. 2), which suggests that GBM is more consistent than BayesB.

### Relative importance of specific FTIR wavelengths for variation in blood metabolites

**Fig. 3:**
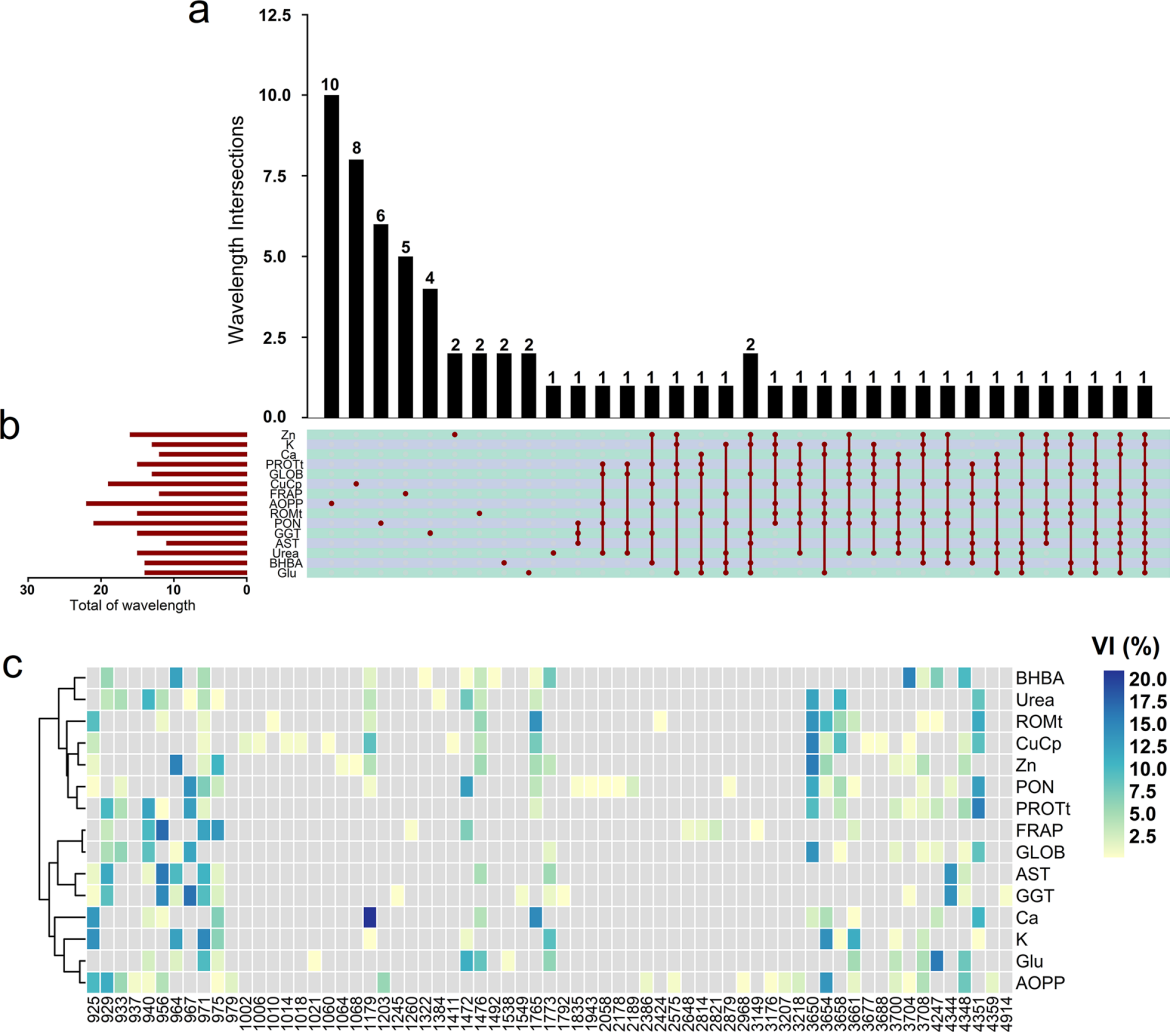
Wavelength regions variable importance (VI) greater than 0.8% for the GBM approach using Model 4 (M4) that includes milk FTIR data, on-farm (DIM and parity), and genomic information for blood metabolites. **a** Overlap of FTIR wavelength considered as significant (VI > 0.8%) indicating specific and shared regions, **b** total of FTIR wavelengths with a relative VI greater than 0.8% of the phenotypic variability and **c** heatmap for relative VI scores for each FTIR wavelength absorbance considered as significant (VI > 0.8%)

The FTIR wavelength regions that captured the phenotypic variation in the GBM approach for phenotype prediction of the blood metabolic profile showed a consistent effect across biological groups (Fig. 3a). In total, 67 wavelengths were identified as the most relevant (i.e., explaining more than 0.8% of the phenotypic variability) with GBM. The number of relevant individual FTIR wavelengths ranged from 11 for AST to 22 for AOPP and covered the three main regions: short-mid wavelength infrared (4351 to 3650 cm^−1^), mid wavelength infrared (1773 to 1179 cm^−1^), and mid-long wavelength infrared (975 to 925 cm^−1^; Fig. 3b). The number of overlapping wavelengths that explained more than 0.8% of the phenotypic variability across the two investigated models (BayesB and GBM) was larger for blood minerals, globulin and RMOt, which shared more than 60% of wavelengths (see Additional file 2: Fig. S3). For urea, AST, PON, AOPP, FRAP and ceruloplasmin, we observed that BayesB and GBM shared from 27 to 42% of wavelengths with the highest contribution for phenotypic prediction (Fig. 3c).

The most significant FTIR wavelengths for the GBM approach (VI > 0.8) are related to overtones and combinations of the vibrations of some chemical bonds, such as C–O symmetric stretching, C=O stretching, C–H, N–H, O–H, and S–H. Twenty-four wavelengths were shared by at least five blood metabolites (Fig. 3a): 971 cm^−1^ (12 metabolites); 975 cm^−1^ (10 metabolites); 925, 3708 and 4348 cm^−1^ (9 metabolites); 929, 940, 956, 1179, 1476, 1765, 3650, and 4351 cm^−1^ (8 metabolites); 964, 1773, 3654, 3658, and 3661 cm^−1^ (7 metabolites); 1472, 3700, 3704, and 4247 cm^−1^ (6 metabolites) and 933 and 967 cm^−1^ (5 metabolites). These shared regions contributed to the predictive ability of GBM, from 0.84% (975 cm^−1^ for urea and 1179 cm^−1^ for K) to 20.96% (1179 cm^−1^ for calcium). In agreement with these results, the major FTIR wavelength was lowly to highly correlated with the evaluated blood metabolites, with values ranging from − 0.75 to − 0.12 and from 0.13 to 0.72 (see Additional file 2: Fig. S4).

## Discussion

### Predictive performance of FTIR data integrated with on-farm and genomic information

In this study, we evaluated the potential of combining FTIR spectroscopy with on-farm and genomic data to predict blood metabolites related to metabolic disorders in Holstein cattle. Such a multi-data approach to predict complex phenotypes is a subject of growing interest, since it better captures their biological meaning and can be exploited to build a prediction model that accurately predicts unknown samples. Including on-farm information (DIM and parity) in the FTIR predictions (Model M2) increased the predictive ability (${\mathrm{R}}^{2}$) with an average RD of 3.7% for energy-related metabolites, 5.1% for liver function/hepatic damage-related metabolites, 4.3% for oxidative stress-related metabolites, 2.8% for inflammation/innate immunity-related metabolites, and 4.6% for minerals (Fig. 1).

The improvements in R^2^ may be related to the direct effect due to lactation stage, which is linked to energy requirements, milk yield, and milk fat and protein concentrations [48]. Metabolic disorders in dairy cows have a direct effect on milk yield and quality [49], which indicates that variations in the milk composition closely reflect alterations in the blood biochemical profile. Within this framework, we observed a slight difference across the DIM classes for blood parameters (see Additional file 1: Table S1 and Additional file 2: Fig. S2), due to the fact that the evaluated cows were within a normal range of physiological values, without visible signs of metabolic disorders [50]. Consistent with this result, a previous study on healthy cows by Premi et al. [34] found non-significant differences between the early (from 28 to 45 DIM) and late (from 160 to 305 DIM) lactation phases for most of the blood metabolites that we considered here. However, substantial differences between DIM classes were observed for urea content, liver function/hepatic damage indicators ROMt, AOPP, ceruloplasmin and zinc (see Additional File 1: Table S1), in agreement with the higher RD observed for M2 (Fig. 1). In addition, Krogh et al. [51] reported that taking herd/diet, parity, and DIM into account does not explain all the variations observed for some blood metabolites (such as, BHBA, NEFA, glucose, and serum IGF-1), which indicates that other factors may contribute to their phenotypic variation.

We found a greater improvement in predictive ability with Model M4, which included FTIR, on-farm, and genomic data, with a RD ranging from 9.1% for inflammation/innate immunity to 16.4% for liver function/hepatic damage (Fig. 1). Including genomic information established a link between genotype and phenotype, which is an effective means of associating the genetic background of an individual with a variation in blood metabolites [47, 52, 53]. By integrating the FTIR predictions with different individual sources of variation (DIM and parity) and genetic backgrounds (SNP data), we reduced the prediction error and increased the R^2^. These different sources of information contribute to better capturing the variation in biological pathways in which the blood metabolites are involved. A potential limitation could be related to the availability of females with genotypic information, although the rapid drop in the price of genotyping has led to a considerable increase in the number of young genotyped females. Therefore, genotyping animals no longer seems to be a limiting factor [54]. An alternative strategy could be to combine milk FTIR spectral information with pedigree data, even if it provides a lower or similar R^2^ compared to the strategy that includes genomic information [29].

Concerning the practical applications of predicting blood metabolites, it is worth noting that the aim of metabolic profiling is not to identify visible diseases, which can be diagnosed with gold standard methods, but to detect subclinical disorders at an early stage to preventively take adequate measures at both the individual and the herd level [55]. Thus, given that phenotyping blood metabolites is invasive, time-consuming, and not feasible at the herd level, prediction from milk samples is very attractive. Moreover, using milk FTIR for large-scale phenotyping of blood metabolic profiles could be exploited by breeding programs that aim at selecting resilient animals [55].

### Predictive performance of the statistical approaches used in this study

We have shown that it is feasible to predict blood metabolite traits in Holstein cattle using different layers of the omics cascade (FTIR and genomic information) and different individual sources of variation (DIM and parity). The multiple sources of information capture the heterogeneity of the biological profiles, creating a complex relationship between the predictor variables, which requires finely-tuned statistical methods to capture the biological variation by identifying the most informative variables [53]. We compared the BayesB and GBM methods for their effectiveness when they include heterogeneous information for phenotypic prediction of the major groups of blood metabolites. Using statistical methods such as Bayesian penalized regression or machine learning algorithms for the prediction of phenotypes makes it possible to select more informative predictor variables capable of improving the predictive ability (${\mathrm{R}}^{2}$) in the validation population and reducing overfitting in the training population. Our results show that GBM outperformed BayesB in predicting blood metabolite-related traits (Tables 3 and 4); the differences in ${\mathrm{R}}^{2}$ between the approaches are due to differences imposed in variable selection, which aim at reducing noise in the training dataset.

The weaker predictive ability of BayesB compared to GBM can be due to considering an additive effect for predictors and a prior assigned as mixture of a peak around zero with a scaled t-distribution [56]. Given this, compared to GBM, BayesB showed predictive abilities that were lower by about 6.07% for energy-related metabolites, 8.54% for liver function/hepatic damage metabolites, 6.78% for oxidative stress metabolites, 4.11% for inflammation/innate immunity metabolites, and 8.24% for minerals. The usefulness of GBM for predicting phenotypes depends on how much this approach accurately models the complex relationships (e.g., non-linear and interaction effects) between predictor variables and/or the target trait [44]. However, the use of automatic variable selection and the possibility of making fewer assumptions regarding phenotype distribution than traditional statistical methods, can help achieve more accurate predictions [41].

Comparing different CV scenarios based on model fit parameters showed that the tenfold CV had a higher predictive ability than the batch-out CV and herd-out CV (Tables 3, 4, 5). The training and validation populations were assigned as random samples in both these CV scenarios, but differences in how the CV scenarios were structured caused differences in relationships between training and validation populations, which affected the model fit parameters. On the one hand, leaving-out 20% of the herd/date data in the batch-out CV scenario compared to the tenfold CV scenario led to a decrease in R^2^ of 11.01% for BayesB and of 7.06% for GBM, which indicates that GBM has a greater adaptive learning rate and a lower reduction in ${\mathrm{R}}^{2}$ than BayesB. On the other hand, considering the greater independence between the training and validation sets in the herd-out CV, we observed a lower accuracy due to the reduction in the ability of predictors to accurately capture the relationship between individuals from the training and validation sets. These reductions in R^2^ observed for the herd-out CV compared to the tenfold CV ranged from 10.59 to 53.05% for Bayes B and from 10.20 to 50.98% for GBM. Qin et al. [57] indicated that random CV under-estimates the error rate of the prediction equation compared with batch-out CV especially when systematic differences exist between batches. Different authors have observed that lower dependencies between training and validation sets led to lower R^2^ and higher prediction errors [25, 58–60]. However, the GBM approach showed greater predictive ability than BayesB, which is due to its power to handle complex scenarios and improve FTIR predictions of blood metabolites with different origins and biological variability.

Among the categories of blood metabolites analyzed, inflammation/innate immunity-related traits had the highest predictive ability in each CV scenario and statistical approach, ranging from 0.52 to 0.82 for ceruloplasmin, from 0.69 to 0.88 for PROTt, and from 0.71 to 0.92 for globulins, these values being higher than those obtained by Luke et al. [55] for globulins (${\mathrm{R}}^{2}$ = 0.12). However, these results are very different from those obtained by Giannuzzi et al. [28] for ceruloplasmin (${\mathrm{R}}^{2}$ = 0.21), PROTt (${\mathrm{R}}^{2}$ = 0.32), and globulins (${\mathrm{R}}^{2}$ = 0.37) who used in-line near-infrared based on 31 light-emitting diodes (LED). Our results suggest that milk FTIR data combined with on-farm and genomic data could be useful for predicting changes in liver function caused by inflammatory events, and could help predict the evolution of the inflammatory response in the medium and long term and understand whether the animals are in a phase of adaptation or chronic stress [34].

Predictive abilities using the M4 Model for energy-related metabolites ranged from 0.63 to 0.80 (BayesB) and from 0.65 to 0.85 (GBM) for glucose, from 0.40 to 0.59 (BayesB) and from 0.43 to 0.62 (GBM) for BHBA, and from0.47 to 0.76 (BayesB) and from 0.49 to 0.80 (GBM) for urea. The values for glucose were higher than those reported by Grelet et al. [18] (${\mathrm{R}}^{2}$ = 0.44) using a fourfold CV and by Benedet et al. [61] (${\mathrm{R}}^{2}$ = 0.20) using a threefold CV, but the prediction accuracy that we obtained for BHBA was lower than that reported by these authors using only FTIR information (0.63 to 0.70), and by Mota et al. [58] using a multi-breed scenario (${\mathrm{R}}^{2}$ = 0.76). However, the predictive ability obtained for BHBA was higher than those reported by Belay et al. [62] (0.46 to 0.66), and Luke et al. [55] (R^2^ from 0.48 to 0.59). These differences between studies may be explained by the differences in the statistical approaches used (PLS regression in the other studies), in the variability of the blood metabolites, and in the DIM in which the blood metabolites were evaluated (i.e., DIM from 5 to 65 were used in the other studies whereas we considered the entire lactation).

Combining on-farm and genomic information and applying statistical approaches for selecting informative FTIR wavelengths, represent key factors for the higher predictive ability observed in our study compared with the results from the aforementioned studies. Energy-related metabolites (glucose, BHBA and urea) have been identified as the major metabolites that are related to the degree of physiological imbalance [1, 63] and deficiencies in protein and energy in the ration [64]. These results indicate that the GBM approach and the full model (M4) could be used to effectively and accurately assess cow energy balance-related traits with the aim of devising strategies to avert impaired energy balance during milk production.

Regarding the blood metabolites related to liver function and hepatic damage, ${\mathrm{R}}^{2}$ values for GGT ranged from 0.45 to 0.63 using BayesB and from 0.48 to 0.65 using GBM, for aspartate aminotransferase (AST) from 0.38 to 0.53 using BayesB and from 0.43 to 0.63 using GBM, and for PON from 0.48 to 0.66 using BayesB and from 0.52 to 0.69 using GBM. The accurate predictions of these metabolites show that hepatic disturbances can be assessed by FTIR with the integration of different sources of biological variation and might be used in detecting changes in liver functions and liver injury in dairy cows. The oxidative stress metabolites exhibited an average predictive ability (${\mathrm{R}}^{2}$) for tenfold and batch-out CV using that ranged approximately from 0.45 to 0.72 for BayesB and from 0.49 to 0.75 for GBM, these values being higher than those reported by Giannuzzi et al. [28] ranging from 0.32 to 0.36. Blood oxidative stress metabolites are related to dysfunctional host immune and inflammatory responses, increasing the cows’ susceptibility to health disorders during the transition phase [65, 66]. In the early postpartum period, the negative energy balance increases lipid mobilization, and lipid β-oxidation in the liver tissue is associated with a greater susceptibility to oxidative stress in dairy cows [2]. Milk infrared spectra accurately predicted blood mineral concentrations in line with previous studies using FTIR to predict milk mineral concentrations, with ${\mathrm{R}}^{2}$ values ranging from 0.33 to 0.87 [67–69].

### Effect of the trait heritability on relative gains in predictive ability between Models 1 and 4 on blood metabolites

The objective of combining FTIR data with multiple sources of information is to make accurate phenotypic predictions to support farm management and breeding decisions. Predictive performance for blood metabolites suggests that the improvement in accuracy depends on the trait analyzed (Fig. 1). The results show that the performance of M4 (integrating FTIR, on-farm, and genomic information) depends highly on the heritability estimates of the traits (Fig. 2). The relationship between the predictive ability of M4 and the heritability of the traits, was, in general, stronger with GBM (${\mathrm{R}}^{2}$ = 0.55, 0.65 and 0.33, depending on the CV scenario) than with BayesB (${\mathrm{R}}^{2}$ = 0.44, 0.36 and 0.16, depending on the CV scenario), even for traits that are mostly polygenic.

### Relative importance of FTIR wavelengths for blood metabolite variation

The aim of blood metabolic profiling is to obtain information on the cows' nutritional status, metabolic health, and resilience [34] within the herd by identifying the prevalence of certain metabolic disorders. FTIR wavelength absorbance could be an effective method for predicting blood metabolites because of its association with milk components, which are indirect indicators of energy balance in dairy cows. Notably, 28 of the 67 wavelengths explaining more than 0.8% of VI were identified as the most relevant wavelengths that explained more than 3% of the blood metabolites' variability and that have a biological link with the main components of milk, such as protein and fatty acids, fat, and pH. In addition, the shared regions between the BayesB and GBM approaches are the three major regions (4351 to 3650 cm^−1^; 1773 to 1179 cm^−1^ and 975 to 925 cm^−1^; Additional file 2: Fig. S3) that interact with the common chemical bonds present in milk components such as fat, protein, lactose, carbohydrates, and organic acids [70–73]. Duffield et al. [74] found that the increase in milk fat and decrease in milk protein concentrations were associated with an elevated BHBA concentration in the serum (sensitivity and specificity of 58% and 69%, respectively), which can explain the observed wavelengths related to chemical bonds interacting with these milk components. De Roos et al. [17] found that the concentration of ketone bodies was associated with milk spectral regions mainly when there were changes in the milk fat and protein contents and in the FA profile during energy-deficient periods, which explains the major contribution of these wavelength regions in accurately predicting blood metabolites in dairy cows.

## Conclusions

This study confirms that, compared to a model that considers only milk FTIR spectral data, combining milk FTIR data with on-farm and genomic information, especially in the case of metabolites under strong genetic control, increased the predictive ability for blood metabolic traits in Holstein cattle. The GBM ensemble approach improved the predictive performance compared to the BayesB model because it extracts a smaller number of informative predictors and captures the non-linear and interaction effects, which lead to greater predictive ability. In addition, compared to BayesB, GBM was less affected when the training and validation sets became more independent. Although further research is required to test the potential of GBM on other populations and breeds, this study provides an integrated statistical approach for the large-scale monitoring of blood indicators of metabolic disorders in dairy cattle for farm management and breeding purposes.

## Supplementary Information

**Additional file 1: Table S1.** Descriptive statistics for blood metabolites. Average and standard deviation (in parentheses) values for blood metabolites across the six classes of days in milk (DIM). **Table S2.** Predictive performances using the tenfold cross-validation scenario. Average milk Fourier transform infrared (FTIR) prediction performance (± SD) for gradient boosting machine (GBM), and BayesB using the tenfold cross-validation scenario considering only the milk FTIR information (M1), the milk FTIR information and on-farm information (DIM and parity; M2) and considering the milk FTIR information and single nucleotide polymorphism (SNP; M3), for blood metabolites. **Table S3.** Predictive performances using the batch-out cross-validation scenario. Average milk Fourier transform infrared (FTIR) prediction performance (± SD) for gradient boosting machine (GBM), and BayesB using the batch-out cross-validation scenario considering only the milk FTIR information (M1), the milk FTIR information and on-farm information (DIM and parity; M2) and considering the milk FTIR information and single nucleotide polymorphism (SNP; M3), for blood metabolites. **Table S4. **Predictive performances using the herd-out cross-validation scenario. Average milk Fourier transform infrared (FTIR) prediction performance (± SD) for gradient boosting machine (GBM), and BayesB using the herd-out cross-validation scenario considering only the milk FTIR information (M1), the milk FTIR information and on-farm information (DIM and parity; M2) and considering the milk FTIR information and single nucleotide polymorphism (SNP; M3), for blood metabolites.

**Additional file 2: Figure S1. **Distribution of the phenotypic values for blood metabolites. Distribution of the phenotypic values for blood metabolites. (a) Energy-related metabolites: BHBA: β-hydroxybutyric acid; GLU: glucose and urea; (b) Liver function/hepatic damage: AST: aspartate aminotransferase, GGT: γ-glutamyl transferase and PON: paraoxonase; (c) Oxidative stress metabolites: AOPP: advanced oxidation protein products, FRAP: ferric reducing antioxidant power, ROMt: total reactive oxygen metabolites; (d) Inflammation/innate immunity: CuCp: ceruloplasmin, GLOB: globulins, PROTt: total proteins; and (e) Minerals: Ca: calcium, K: potassium, and Zn: zinc. **Figure S2. **oxplot of the phenotypic values for blood metabolites. Boxplot of the phenotypic values for blood metabolites across the six classes of days in milk (DIM). (a) Energy-related metabolites: BHBA: β-hydroxybutyric acid; GLU: glucose and urea; (b) Liver function/hepatic damage: AST: aspartate aminotransferase, GGT: γ-glutamyl transferase and PON: paraoxonase; (c) Oxidative stress metabolites: AOPP: advanced oxidation protein products, FRAP: ferric reducing antioxidant power, ROMt: total reactive oxygen metabolites; (d) Inflammation/innate immunity: CuCp: ceruloplasmin, GLOB: globulins, PROTt: total proteins; and (e) Minerals: Ca: calcium, K: potassium, and Zn: zinc. Class of days in milk (DIM): CL1: less than 60 days; CL 2: from 60 to 120 days; CL 3: from 121 to 180 days; CL 4: from 181 to 240 days; CL 5: from 241 to 300 days; and CL 6: higher than > 300 days. **Figure S3. **Venn diagrams of the most informative milk FTIR wavelengths for blood metabolites using BayesB and gradient boosting machine (GBM). Venn diagrams showing the unique and shared milk FTIR wavelengths explaining more than 0.8% of the phenotypic variability for BayesB and gradient boosting machine (GBM) for blood metabolites. (a) Energy-related metabolites: BHBA: β-hydroxybutyric acid; GLU: glucose and urea; (b) Liver function/hepatic damage: AST: aspartate aminotransferase, GGT: γ-glutamyl transferase and: paraoxonase; (c) Oxidative stress metabolites: AOPP: advanced oxidation protein products, FRAP: ferric reducing antioxidant power, ROMt: total reactive oxygen metabolites; (d) Inflammation/innate immunity: CuCp: ceruloplasmin, GLOB: globulins, PROTt: total proteins; and (e) Minerals: Ca: calcium, K: potassium, and Zn: zinc. **Figure S4. **Pearson correlation of most informative wavelength regions with blood metabolites. Pearson correlation between wavelength regions explains more than 0.8% of the phenotypic variability in the GBM approach and blood metabolites in the Holstein cow population. AOPP: advanced oxidation protein products; AST: aspartate aminotransferase; BHBA: β-hydroxybutyric acid; Ca: calcium; CuCp: ceruloplasmin; FRAP: ferric reducing antioxidant power; GGT: γ-glutamyl transferase; GLOB:globulins; GLU: glucose; K: potassium; PON: paraoxonase; PROTt: total proteins; ROMt: total reactive oxygen metabolites; Zn: zinc.

## Data Availability

The phenotypic and genotypic information are available for academic use from the authors upon reasonable request (contacting the researcher Alessio Cecchinato e-mail: alessio.cecchinato@unipd.it).
