# Supplementary material for: Integrating on-farm and genomic information improves the predictive ability of milk infrared prediction of blood indicators of metabolic disorders in dairy cows
Source: Genet Sel Evol. 2023 Apr 3;55:23. doi: 10.1186/s12711-023-00795-1 (PMC10069109; doi:10.1186/s12711-023-00795-1)
Supplement: Supplementary file 1 — Additional file 1: Table S1. Descriptive statistics for blood metabolites. Average and standard deviation (in parentheses) values for blood metabolites across the six classes of days in milk (DIM). Table S2. Predictive performances using the tenfold cross-validation scenario. Average milk Fourier transform infrared (FTIR) prediction performance (± SD) for gradient boosting machine (GBM), and BayesB using the tenfold cross-validation scenario considering only the milk FTIR information (M1), the milk FTIR information and on-farm information (DIM and parity; M2) and considering the milk FTIR information and single nucleotide polymorphism (SNP; M3), for blood metabolites. Table S3. Predictive performances using the batch-out cross-validation scenario. Average milk Fourier transform infrared (FTIR) prediction performance (± SD) for gradient boosting machine (GBM), and BayesB using the batch-out cross-validation scenario considering only the milk FTIR information (M1), the milk FTIR information and on-farm information (DIM and parity; M2) and considering the milk FTIR information and single nucleotide polymorphism (SNP; M3), for blood metabolites. Table S4. Predictive performances using the herd-out cross-validation scenario. Average milk Fourier transform infrared (FTIR) prediction performance (± SD) for gradient boosting machine (GBM), and BayesB using the herd-out cross-validation scenario considering only the milk FTIR information (M1), the milk FTIR information and on-farm information (DIM and parity; M2) and considering the milk FTIR information and single nucleotide polymorphism (SNP; M3), for blood metabolites. [file 12711_2023_795_MOESM1_ESM.docx]

**Integrating on-farm and genomic information improves the predictive ability of milk infrared prediction of blood indicators of metabolic disorders in dairy cows**

Lucio F. M. Mota^1*^, Diana Giannuzzi^1^, Sara Pegolo^1^, Erminio Trevisi^2,3^, Paolo Ajmone Marsan^2,3^, Alessio Cecchinato^1^

^1^Department of Agronomy, Food, Natural Resources, Animals and Environment (DAFNAE), University of Padova, 35020, Legnaro (PD), Italy

^2^Department of Animal Science, Food and Nutrition (DIANA) and the Romeo and Enrica Invernizzi Research Center for Sustainable Dairy Production (CREI), Faculty of Agricultural, Food, and Environmental Sciences, Università Cattolica del Sacro Cuore, 29122, Piacenza, Italy

^3^Nutrigenomics and Proteomics Research Center, Università Cattolica del Sacro Cuore, 29122, Piacenza, Italy

*Corresponding author:

Lucio F M Mota, e-mail: [flaviommota.zoo@gmail.com](mailto:flaviommota.zoo@gmail.com)

# Additional File 1

**Table S1** Average and its standard deviation values (in parentheses) for blood metabolites across the six classes of days in milk (DIM).

| **Trait^1^** | **Classes of Days in Milk^2^** | | | | | |
| --- | --- | --- | --- | --- | --- | --- |
|  | **CL 1** | **CL 2** | **CL 3** | **CL 4** | **CL 5** | **CL 6** |
| Energy-related metabolites | | | | | | |
| Glucose, mmol/l | 4.32 ± 0.42 | 4.36 ± 0.34 | 4.35 ± 0.31 | 4.31 ± 0.29 | 4.31 ± 0.3 | 4.45 ± 0.28 |
| BHBA, mmol/l | 0.54 ± 0.19 | 0.53 ± 0.17 | 0.50 ± 0.15 | 0.54 ± 0.17 | 0.55 ± 0.17 | 0.55 ± 0.17 |
| Urea, mmol/l | 5.86 ± 1.03 | 6.58 ± 0.99 | 6.72 ± 1.10 | 6.39 ± 0.89 | 6.39 ± 1.12 | 6.27 ± 0.91 |
| Liver function/hepatic damage | | | | | | |
| AST, U/l | 93.28 ± 16.73 | 95.88 ± 17.05 | 107.04 ± 23.06 | 103.16 ± 22.62 | 104.31 ± 24.62 | 97.26 ± 20.45 |
| GGT, U/l | 21.51 ± 5.12 | 26.81 ± 6.79 | 29.85 ± 6.79 | 30.43 ± 7.07 | 30.21 ± 6.73 | 29.7 ± 6.04 |
| PON, U/ml | 101.65 ± 22.68 | 105.76 ± 19.53 | 98.55 ± 18.12 | 94.36 ± 18.75 | 92.69 ± 17.49 | 89.7 ± 13.77 |
| Oxidative stress metabolites | | | | | | |
| ROMt, mgH_2_O_2_/100ml | 13.16 ± 2.83 | 12.03 ± 3.20 | 12.12 ± 3.18 | 12.55 ± 2.98 | 12.9 ± 2.82 | 13.91 ± 2.95 |
| AOPP, µmol/l | 40.75 ± 8.30 | 47.38 ± 8.83 | 48.86 ± 7.54 | 48.79 ± 8.77 | 50.07 ± 8.70 | 51.07 ± 9.80 |
| FRAP, µmol/l | 188.03 ± 35.29 | 194.5 ± 38.47 | 201.67 ± 35.5 | 199.27 ± 39.08 | 196.05 ± 33.22 | 193.73 ± 37.19 |
| Inflammation / innate immunity | | | | | | |
| Ceruloplasmin, µmol/l | 2.00 ± 0.60 | 1.71 ± 0.57 | 1.73 ± 0.58 | 1.88 ± 0.54 | 2.00 ± 0.58 | 2.18 ± 0.59 |
| PROTt, g/l | 79.67 ± 4.82 | 81.48 ± 4.7 | 82.05 ± 4.54 | 81.48 ± 4.51 | 81.46 ± 5.22 | 80.78 ± 4.81 |
| Globulins, g/l | 43.4 ± 4.67 | 44.1 ± 5.52 | 44.23 ± 5.28 | 43.85 ± 5.28 | 43.73 ± 5.8 | 42.92 ± 5.22 |
| Minerals | | | | | | |
| Calcium, mmol/l | 2.49 ± 0.12 | 2.53 ± 0.12 | 2.53 ± 0.11 | 2.50 ± 0.11 | 2.52 ± 0.10 | 2.48 ± 0.10 |
| Potassium, mmol/l | 3.99 ± 0.42 | 4.15 ± 0.38 | 4.24 ± 0.40 | 4.12 ± 0.41 | 4.16 ± 0.40 | 4.08 ± 0.36 |
| Zinc, µmol/l | 11.68 ± 3.20 | 11.44 ± 2.60 | 11.73 ± 2.23 | 12.02 ± 2.44 | 12.28 ± 2.37 | 13.19 ± 2.88 |

^1^BHBA = β-hydroxybutyric acid; AST = aspartate aminotransferase; GGT = γ-glutamyl transferase; PON = paraoxonase; ROMt = total reactive oxygen metabolites; AOPP = advanced oxidation protein products; FRAP = ferric reducing antioxidant power; PROTt = total proteins.

^2^ Classes of Days in Milk: CL1 - less than 60 days; CL 2 - from 60 to 120 days; CL 3 - from 121 to 180 days; CL 4 - from 181 to 240 days; CL 5, - from 241 to 300 days; CL 6 – higher than >300 days.

**Table S2** Average milk Fourier transform infrared (FTIR) prediction performance (± SD) for gradient boosting machine (GBM), and Bayesian B (BayesB) using the 10-folds cross-validation considering only the milk FTIR information (M1), the milk FTIR information and on-farm information (DIM and parity; M2) and considering the milk FTIR information and single nucleotide polymorphism (SNP; M3), for blood metabolites.

| **Trait^1^** | **Model** | **Method** | | | | | | |
| --- | --- | --- | --- | --- | --- | --- | --- | --- |
|  |  | **BayesB** | | |  | **GBM** | | |
|  |  | **R^2^** | **RMSE** | **Slope** |  | **R^2^** | **RMSE** | **Slope** |
| *Energy-related metabolites* | | | | | | | | |
| Glucose, mmol/l | FTIR (M1) | 0.72 ± 0.066 | 0.17 ± 0.013 | 1.08 ± 0.099 |  | 0.75 ± 0.038 | 0.15 ± 0.010 | 0.98 ± 0.077 |
|  | FTIR + on farm (M2) | 0.75 ± 0.047 | 0.16 ± 0.022 | 1.02 ± 0.090 |  | 0.79 ± 0.040 | 0.14 ± 0.010 | 1.02 ± 0.080 |
|  | FTIR + SNP (M3) | 0.77 ± 0.047 | 0.16 ± 0.009 | 1.01 ± 0.095 |  | 0.81 ± 0.043 | 0.13 ± 0.062 | 1.07 ± 0.059 |
| BHBA, mmol/l | FTIR (M1) | 0.54 ± 0.072 | 0.15 ± 0.010 | 0.96 ± 0.125 |  | 0.57 ± 0.078 | 0.12 ± 0.011 | 1.03 ± 0.096 |
|  | FTIR + on farm (M2) | 0.56 ± 0.060 | 0.13 ± 0.009 | 1.02 ± 0.113 |  | 0.59 ± 0.046 | 0.12 ± 0.031 | 0.98 ± 0.075 |
|  | FTIR + SNP (M3) | 0.57 ± 0.061 | 0.12 ± 0.006 | 1.01 ± 0.139 |  | 0.61 ± 0.040 | 0.10 ± 0.010 | 1.09 ± 0.096 |
| Urea, mmol/l | FTIR (M1) | 0.71 ± 0.055 | 0.54 ± 0.037 | 1.01 ± 0.100 |  | 0.73 ± 0.045 | 0.54 ± 0.039 | 1.01 ± 0.073 |
|  | FTIR + on farm (M2) | 0.73 ± 0.047 | 0.56 ± 0.037 | 1.01 ± 0.097 |  | 0.76 ± 0.041 | 0.52 ± 0.034 | 0.99 ± 0.069 |
|  | FTIR + SNP (M3) | 0.75 ± 0.054 | 0.57 ± 0.039 | 1.01 ± 0.090 |  | 0.78 ± 0.047 | 0.89 ± 0.164 | 1.08 ± 0.064 |
| *Liver function/hepatic damage* | | | | | | | | |
| AST, U/l | FTIR (M1) | 0.45 ± 0.144 | 15.91 ± 3.275 | 1.13 ± 0.210 |  | 0.55 ± 0.060 | 14.79 ± 1.075 | 0.95 ± 0.101 |
|  | FTIR + on farm (M2) | 0.47 ± 0.094 | 14.21 ± 1.348 | 1.01 ± 0.157 |  | 0.57 ± 0.079 | 13.78 ± 1.068 | 1.01 ± 0.090 |
|  | FTIR + SNP (M3) | 0.49 ± 0.089 | 13.72 ± 1.263 | 1.02 ± 0.149 |  | 0.59 ± 0.064 | 13.1 ± 1.063 | 0.98 ± 0.081 |
| GGT, U/l | FTIR (M1) | 0.54 ± 0.070 | 4.86 ± 0.384 | 1.07 ± 0.101 |  | 0.56 ± 0.060 | 4.77 ± 0.301 | 1.04 ± 0.093 |
|  | FTIR + on farm (M2) | 0.56 ± 0.064 | 4.82 ± 0.369 | 0.99 ± 0.097 |  | 0.59 ± 0.080 | 4.55 ± 0.42 | 1.09 ± 0.091 |
|  | FTIR + SNP (M3) | 0.58 ± 0.058 | 4.07 ± 0.470 | 1.02 ± 0.057 |  | 0.61 ± 0.064 | 4.27 ± 0.975 | 0.95 ± 0.084 |
| PON, U/ml | FTIR (M1) | 0.57 ± 0.076 | 12.73 ± 1.158 | 1.03 ± 0.116 |  | 0.59 ± 0.040 | 12.37 ± 0.990 | 1.02 ± 0.101 |
|  | FTIR + on farm (M2) | 0.60 ± 0.039 | 12.01 ± 1.188 | 1.01 ± 0.112 |  | 0.62 ± 0.030 | 11.69 ± 0.950 | 1.01 ± 0.090 |
|  | FTIR + SNP (M3) | 0.62 ± 0.054 | 11.83 ± 1.107 | 1.07 ± 0.125 |  | 0.64 ± 0.034 | 11.63 ± 1.037 | 1.03 ± 0.053 |
| *Oxidative stress metabolites* | | | | | | | | |
| ROMt, mgH_2_O_2_/100ml | FTIR (M1) | 0.73 ± 0.066 | 1.57 ± 0.283 | 1.07 ± 0.069 |  | 0.75 ± 0.030 | 1.50 ± 0.190 | 1.01 ± 0.069 |
|  | FTIR + on farm (M2) | 0.75 ± 0.062 | 1.51 ± 0.313 | 0.99 ± 0.053 |  | 0.77 ± 0.040 | 1.46 ± 0.220 | 1.02 ± 0.052 |
|  | FTIR + SNP (M3) | 0.77 ± 0.042 | 1.48 ± 0.184 | 1.00 ± 0.06 |  | 0.79 ± 0.041 | 1.43 ± 0.157 | 0.97 ± 0.057 |
| AOPP, µmol/l | FTIR (M1) | 0.61 ± 0.067 | 5.72 ± 0.553 | 1.02 ± 0.075 |  | 0.63 ± 0.050 | 5.49 ± 0.467 | 1.09 ± 0.090 |
|  | FTIR + on farm (M2) | 0.63 ± 0.057 | 5.66 ± 0.51 | 1.00 ± 0.079 |  | 0.66 ± 0.050 | 5.18 ± 0.489 | 1.06 ± 0.089 |
|  | FTIR + SNP (M3) | 0.66 ± 0.063 | 5.31 ± 0.491 | 1.03 ± 0.061 |  | 0.69 ± 0.188 | 5.02 ± 0.316 | 0.93 ± 0.481 |
| FRAP, µmol/l | FTIR (M1) | 0.49 ± 0.068 | 27.90 ± 2.905 | 1.11 ± 0.094 |  | 0.51 ± 0.090 | 26.40 ± 2.370 | 1.07 ± 0.079 |
|  | FTIR + on farm (M2) | 0.51 ± 0.059 | 26.95 ± 1.559 | 1.07 ± 0.084 |  | 0.53 ± 0.060 | 25.15 ± 1.461 | 1.09 ± 0.068 |
|  | FTIR + SNP (M3) | 0.52 ± 0.084 | 25.98 ± 1.914 | 1.15 ± 0.142 |  | 0.55 ± 0.086 | 24.96 ± 1.582 | 0.96 ± 0.074 |
| *Inflammation/innate immunity* | | | | | | | | |
| Ceruloplasmin, µmol/l | FTIR (M1) | 0.70 ± 0.056 | 0.33 ± 0.047 | 1.07 ± 0.134 |  | 0.72 ± 0.040 | 0.32 ± 0.030 | 1.03 ± 0.060 |
|  | FTIR + on farm (M2) | 0.73 ± 0.052 | 0.32 ± 0.056 | 1.06 ± 0.128 |  | 0.76 ± 0.037 | 0.30 ± 0.028 | 1.05 ± 0.060 |
|  | FTIR + SNP (M3) | 0.75 ± 0.058 | 0.31 ± 0.043 | 1.01 ± 0.13 |  | 0.78 ± 0.185 | 0.29 ± 0.081 | 0.97 ± 0.056 |
| PROTt, g/l | FTIR (M1) | 0.79 ± 0.039 | 2.15 ± 0.169 | 1.09 ± 0.056 |  | 0.81 ± 0.040 | 2.02 ± 0.158 | 1.03 ± 0.040 |
|  | FTIR + on farm (M2) | 0.80 ± 0.032 | 2.09 ± 0.160 | 0.97 ± 0.044 |  | 0.83 ± 0.030 | 1.97 ± 0.137 | 1.02 ± 0.050 |
|  | FTIR + SNP (M3) | 0.83 ± 0.042 | 1.98 ± 0.154 | 1.05 ± 0.06 |  | 0.85 ± 0.211 | 1.95 ± 1.316 | 1.03 ± 0.049 |
| Globulins, g/l | FTIR (M1) | 0.84 ± 0.024 | 2.04 ± 0.180 | 0.95 ± 0.042 |  | 0.85 ± 0.030 | 1.95 ± 0.165 | 1.02 ± 0.070 |
|  | FTIR + on farm (M2) | 0.86 ± 0.024 | 1.91 ± 0.210 | 1.05 ± 0.040 |  | 0.88 ± 0.030 | 1.78 ± 0.180 | 1.02 ± 0.030 |
|  | FTIR + SNP (M3) | 0.87 ± 0.022 | 1.85 ± 0.172 | 1.02 ± 0.036 |  | 0.89± 0.044 | 1.75 ± 0.105 | 0.97 ± 0.056 |
| *Minerals* | | | | | | | | |
| Calcium, mmol/l | FTIR (M1) | 0.55 ± 0.065 | 0.08 ± 0.004 | 0.96 ± 0.103 |  | 0.58 ± 0.055 | 0.07 ± 0.010 | 0.98 ± 0.080 |
|  | FTIR + on farm (M2) | 0.57 ± 0.068 | 0.07 ± 0.005 | 0.99 ± 0.108 |  | 0.60 ± 0.047 | 0.06 ± 0.007 | 1.00 ± 0.093 |
|  | FTIR + SNP (M3) | 0.59 ± 0.083 | 0.07 ± 0.006 | 0.98 ± 0.119 |  | 0.65 ± 0.083 | 0.06 ± 0.016 | 0.99 ± 0.089 |
| Potassium, mmol/l | FTIR (M1) | 0.61 ± 0.076 | 0.25 ± 0.018 | 1.07 ± 0.105 |  | 0.64 ± 0.039 | 0.23 ± 0.016 | 1.02 ± 0.062 |
|  | FTIR + on farm (M2) | 0.64 ± 0.063 | 0.24 ± 0.019 | 1.05 ± 0.092 |  | 0.66 ± 0.030 | 0.23 ± 0.022 | 0.97 ± 0.065 |
|  | FTIR + SNP (M3) | 0.65 ± 0.076 | 0.25 ± 0.017 | 1.01 ± 0.117 |  | 0.68 ± 0.076 | 0.22 ± 0.020 | 0.98 ± 0.075 |
| Zinc, µmol/l | FTIR (M1) | 0.56 ± 0.053 | 1.75 ± 0.071 | 1.07 ± 0.116 |  | 0.60 ± 0.050 | 1.68 ± 0.066 | 1.06 ± 0.091 |
|  | FTIR + on farm (M2) | 0.60 ± 0.036 | 1.67 ± 0.049 | 1.06 ± 0.098 |  | 0.65 ± 0.040 | 1.55 ± 0.070 | 1.04 ± 0.089 |
|  | FTIR + SNP (M3) | 0.61 ± 0.059 | 1.65 ± 0.107 | 1.02 ± 0.085 |  | 0.67 ± 0.045 | 1.52 ± 0.087 | 0.98 ± 0.070 |

^1^BHBA = β-hydroxybutyric acid; AST = aspartate aminotransferase; GGT = γ-glutamyl transferase; PON = paraoxonase; ROMt = total reactive oxygen metabolites; AOPP = advanced oxidation protein products; FRAP = ferric reducing antioxidant power; PROTt = total proteins. R^2^ – coefficient of determination between the observed and predicted phenotypes in validation set and standard deviation (SD) as the variability measurement of predictive ability; RMSE – root mean squared error.

**Table S3** Average milk Fourier transform infrared (FTIR) prediction performance (± SD) for gradient boosting machine (GBM), and Bayesian B (BayesB) using the batch-out cross-validation considering only the milk FTIR information (M1), the milk FTIR information and on-farm information (DIM and parity; M2) and considering the milk FTIR information and single nucleotide polymorphism (SNP; M3), for blood metabolites.

| **Trait^1^** | **Model** | **Method** | | | | | | |
| --- | --- | --- | --- | --- | --- | --- | --- | --- |
|  |  | **BayesB** | | |  | **GBM** | | |
|  |  | **R^2^** | **RMSE** | **Slope** |  | **R^2^** | **RMSE** | **Slope** |
| *Energy-related metabolites* | | | | | | | | |
| Glucose, mmol/l | FTIR (M1) | 0.68 ± 0.056 | 0.19 ± 0.013 | 1.05 ± 0.093 |  | 0.70 ± 0.044 | 0.18 ± 0.007 | 1.01 ± 0.082 |
|  | FTIR + on farm (M2) | 0.70 ± 0.048 | 0.18 ± 0.013 | 1.04 ± 0.109 |  | 0.74 ± 0.029 | 0.17 ± 0.006 | 1.01 ± 0.058 |
|  | FTIR + SNP (M3) | 0.72 ± 0.049 | 0.17 ± 0.012 | 1.01 ± 0.059 |  | 0.76 ± 0.040 | 0.16 ± 0.011 | 0.97 ± 0.069 |
| BHBA, mmol/l | FTIR (M1) | 0.49 ± 0.059 | 0.16 ± 0.015 | 1.07 ± 0.171 |  | 0.52 ± 0.060 | 0.12 ± 0.007 | 0.99 ± 0.082 |
|  | FTIR + on farm (M2) | 0.50 ± 0.055 | 0.14 ± 0.018 | 1.04 ± 0.576 |  | 0.54 ± 0.076 | 0.12 ± 0.007 | 1.01 ± 0.0636 |
|  | FTIR + SNP (M3) | 0.51 ± 0.049 | 0.13 ± 0.008 | 1.06 ± 0.14 |  | 0.56 ± 0.049 | 0.11 ± 0.006 | 1.02 ± 0.084 |
| Urea, mmol/l | FTIR (M1) | 0.59 ± 0.072 | 0.62 ± 0.065 | 1.02 ± 0.118 |  | 0.67 ± 0.055 | 0.55 ± 0.043 | 1.02 ± 0.077 |
|  | FTIR + on farm (M2) | 0.61 ± 0.054 | 0.59 ± 0.046 | 1.02 ± 0.105 |  | 0.70 ± 0.056 | 0.51 ± 0.046 | 1.03 ± 0.078 |
|  | FTIR + SNP (M3) | 0.63 ± 0.082 | 0.56 ± 0.091 | 0.9 ± 0.091 |  | 0.72 ± 0.082 | 0.50 ± 0.061 | 0.97 ± 0.075 |
| *Liver function/hepatic damage* | | | | | | | | |
| AST, U/l | FTIR (M1) | 0.45 ± 0.116 | 17.25 ± 1.261 | 0.96 ± 0.156 |  | 0.53 ± 0.100 | 15.96 ± 1.164 | 1.04 ± 0.153 |
|  | FTIR + on farm (M2) | 0.47 ± 0.103 | 16.96 ± 2.042 | 1.14 ± 0.319 |  | 0.57 ± 0.065 | 15.58 ± 0.595 | 1.02 ± 0.148 |
|  | FTIR + SNP (M3) | 0.49 ± 0.072 | 15.94 ± 1.006 | 1.08 ± 0.230 |  | 0.58 ± 0.068 | 15.04 ± 1.056 | 0.98 ± 0.083 |
| GGT, U/l | FTIR (M1) | 0.47 ± 0.079 | 5.15 ± 0.552 | 0.93 ± 0.116 |  | 0.52 ± 0.051 | 4.92 ± 0.277 | 1.03 ± 0.138 |
|  | FTIR + on farm (M2) | 0.49 ± 0.038 | 5.10 ± 0.285 | 1.08 ± 0.191 |  | 0.55 ± 0.051 | 4.67 ± 0.221 | 1.03 ± 0.133 |
|  | FTIR + SNP (M3) | 0.51 ± 0.05 | 5.09 ± 0.217 | 0.95 ± 0.122 |  | 0.57 ± 0.05 | 4.69 ± 0.217 | 0.95 ± 0.112 |
| PON, U/ml | FTIR (M1) | 0.52 ± 0.072 | 13.5 ± 1.089 | 0.98 ± 0.121 |  | 0.55 ± 0.054 | 12.91 ± 0.864 | 1.01 ± 0.073 |
|  | FTIR + on farm (M2) | 0.54 ± 0.071 | 12.55 ± 1.156 | 1.02 ± 0.098 |  | 0.59± 0.053 | 12.43 ± 0.829 | 1.05 ± 0.067 |
|  | FTIR + SNP (M3) | 0.56 ± 0.088 | 13.6 ± 1.462 | 1.08 ± 0.087 |  | 0.61 ± 0.049 | 12.36 ± 1.462 | 0.97 ± 0.087 |
| *Oxidative stress metabolites* | | | | | | | | |
| ROMt, mgH_2_O_2_/100ml | FTIR (M1) | 0.68 ± 0.047 | 1.69 ± 0.18 | 0.98 ± 0.098 |  | 0.70 ± 0.036 | 1.61 ± 0.096 | 1.02 ± 0.078 |
|  | FTIR + on farm (M2) | 0.70 ± 0.045 | 1.65 ± 0.11 | 1.01 ± 0.123 |  | 0.72 ± 0.031 | 1.52 ± 0.090 | 1.01 ± 0.085 |
|  | FTIR + SNP (M3) | 0.71 ± 0.061 | 1.64 ± 0.159 | 1.02 ± 0.113 |  | 0.74 ± 0.041 | 1.50 ± 0.159 | 1.01 ± 0.113 |
| AOPP, µmol/l | FTIR (M1) | 0.50 ± 0.059 | 6.59 ± 0.968 | 1.01 ± 0.170 |  | 0.58 ± 0.079 | 5.75 ± 0.416 | 0.98 ± 0.121 |
|  | FTIR + on farm (M2) | 0.52 ± 0.06 | 6.12 ± 0.953 | 1.02 ± 0.131 |  | 0.60 ± 0.083 | 5.68 ± 0.333 | 0.99 ± 0.144 |
|  | FTIR + SNP (M3) | 0.53 ± 0.06 | 6.00 ± 0.905 | 1.02 ± 0.188 |  | 0.61 ± 0.060 | 5.65 ± 0.556 | 0.97 ± 0.098 |
| FRAP, µmol/l | FTIR (M1) | 0.38 ± 0.040 | 28.43 ± 5.18 | 1.17 ± 0.318 |  | 0.42 ± 0.071 | 25.37 ± 1.724 | 0.99 ± 0.160 |
|  | FTIR + on farm (M2) | 0.39 ± 0.034 | 25.72 ± 2.289 | 1.10 ± 0.153 |  | 0.44 ± 0.077 | 23.6 ± 1.610 | 1.02 ± 0.157 |
|  | FTIR + SNP (M3) | 0.40 ± 0.034 | 25.53 ± 5.021 | 0.92 ± 0.286 |  | 0.46 ± 0.134 | 26.53 ± 5.021 | 0.95 ± 0.106 |
| *Inflammation/innate immunity* | | | | | | | | |
| Ceruloplasmin, µmol/l | FTIR (M1) | 0.63 ± 0.076 | 0.34 ± 0.028 | 1.08 ± 0.114 |  | 0.66 ± 0.064 | 0.32 ± 0.019 | 1.01 ± 0.098 |
|  | FTIR + on farm (M2) | 0.65 ± 0.070 | 0.33 ± 0.027 | 1.01 ± 0.097 |  | 0.69 ± 0.067 | 0.32 ± 0.02 | 1.01 ± 0.099 |
|  | FTIR + SNP (M3) | 0.66 ± 0.084 | 0.35 ± 0.031 | 0.98 ± 0.112 |  | 0.71 ± 0.058 | 0.32 ± 0.016 | 0.99 ± 0.082 |
| PROTt, g/l | FTIR (M1) | 0.73 ± 0.066 | 2.49 ± 0.469 | 0.97 ± 0.034 |  | 0.78 ± 0.021 | 2.22 ± 0.188 | 1.02 ± 0.047 |
|  | FTIR + on farm (M2) | 0.75 ± 0.057 | 2.23 ± 0.225 | 1.01 ± 0.064 |  | 0.81 ± 0.019 | 2.02 ± 0.153 | 1.05 ± 0.062 |
|  | FTIR + SNP (M3) | 0.76 ± 0.019 | 2.14 ± 0.189 | 0.97 ± 0.057 |  | 0.83 ± 0.019 | 2.01 ± 0.125 | 0.99 ± 0.042 |
| Globulins, g/l | FTIR (M1) | 0.79 ± 0.047 | 2.37 ± 0.215 | 0.93 ± 0.032 |  | 0.81 ± 0.026 | 2.21 ± 0.18 | 1.03 ± 0.053 |
|  | FTIR + on farm (M2) | 0.80 ± 0.038 | 2.14 ± 0.204 | 1.04 ± 0.062 |  | 0.82 ± 0.018 | 2.08 ± 0.166 | 0.99 ± 0.046 |
|  | FTIR + SNP (M3) | 0.81 ± 0.022 | 2.1 ± 0.12 | 0.97 ± 0.044 |  | 0.84 ± 0.022 | 2.1 ± 0.12 | 0.97 ± 0.044 |
| *Minerals* | | | | | | | | |
| Calcium, mmol/l | FTIR (M1) | 0.48 ± 0.036 | 0.08 ± 0.006 | 0.94 ± 0.077 |  | 0.56 ± 0.032 | 0.07 ± 0.003 | 1.03 ± 0.071 |
|  | FTIR + on farm (M2) | 0.50 ± 0.039 | 0.08 ± 0.004 | 1.02 ± 0.084 |  | 0.58 ± 0.035 | 0.07 ± 0.003 | 1.01 ± 0.079 |
|  | FTIR + SNP (M3) | 0.51 ± 0.040 | 0.08 ± 0.005 | 0.93 ± 0.078 |  | 0.60 ± 0.04 | 0.07 ± 0.002 | 0.97 ± 0.068 |
| Potassium, mmol/l | FTIR (M1) | 0.54 ± 0.083 | 0.27 ± 0.013 | 0.98 ± 0.094 |  | 0.58 ± 0.088 | 0.25 ± 0.009 | 0.99 ± 0.088 |
|  | FTIR + on farm (M2) | 0.55 ± 0.079 | 0.25 ± 0.008 | 1.02 ± 0.119 |  | 0.62 ± 0.084 | 0.24 ± 0.011 | 0.99 ± 0.089 |
|  | FTIR + SNP (M3) | 0.56 ± 0.082 | 0.27 ± 0.014 | 1.03 ± 0.075 |  | 0.64 ± 0.062 | 0.24 ± 0.010 | 1.03 ± 0.080 |
| Zinc, µmol/l | FTIR (M1) | 0.51 ± 0.080 | 1.78 ± 0.301 | 1.08 ± 0.314 |  | 0.57 ± 0.088 | 1.54 ± 0.063 | 0.98 ± 0.114 |
|  | FTIR + on farm (M2) | 0.53 ± 0.078 | 1.67 ± 0.240 | 0.97 ± 0.217 |  | 0.62 ± 0.085 | 1.52 ± 0.243 | 0.96 ± 0.184 |
|  | FTIR + SNP (M3) | 0.55 ± 0.044 | 1.95 ± 0.416 | 0.95 ± 0.261 |  | 0.65 ± 0.054 | 1.50 ± 0.206 | 1.02 ± 0.091 |

^1^BHBA = β-hydroxybutyric acid; AST = aspartate aminotransferase; GGT = γ-glutamyl transferase; PON = paraoxonase; ROMt = total reactive oxygen metabolites; AOPP = advanced oxidation protein products; FRAP = ferric reducing antioxidant power; PROTt = total proteins. R^2^ – coefficient of determination between the observed and predicted phenotypes in validation set and standard deviation (SD) as the variability measurement of predictive ability; RMSE – root mean squared error.

**Table S4** Average milk Fourier transform infrared (FTIR) prediction performance (± SD) for gradient boosting machine (GBM), and Bayesian B (BayesB) using the herd-out cross-validation considering only the milk FTIR information (M1), the milk FTIR information and on-farm information (DIM and parity; M2) and considering the milk FTIR information and single nucleotide polymorphism (SNP; M3), for blood metabolites.

| **Trait^1^** | **Model** | **Method** | | | | | | |
| --- | --- | --- | --- | --- | --- | --- | --- | --- |
|  |  | **BayesB** | | |  | **GBM** | | |
|  |  | **R^2^** | **RMSE** | **Slope** |  | **R^2^** | **RMSE** | **Slope** |
| *Energy-related metabolites* | | | | | | | | |
| Glucose, mmol/l | FTIR (M1) | 0.55 ± 0.085 | 0.25 ± 0.033 | 1.12 ± 0.107 |  | 0.57 ± 0.099 | 0.21 ± 0.029 | 1.08 ± 0.096 |
|  | FTIR + on farm (M2) | 0.56 ± 0.080 | 0.22 ± 0.05 | 1.10 ± 0.116 |  | 0.59 ± 0.096 | 0.19 ± 0.033 | 0.96 ± 0.094 |
|  | FTIR + SNP (M3) | 0.58 ± 0.079 | 0.20 ± 0.074 | 1.12 ± 0.072 |  | 0.60 ± 0.091 | 0.18 ± 0.064 | 1.09 ± 0.063 |
| BHBA, mmol/l | FTIR (M1) | 0.35 ± 0.095 | 0.21 ± 0.017 | 1.07 ± 0.017 |  | 0.38 ± 0.088 | 0.18 ± 0.005 | 1.07 ± 0.015 |
|  | FTIR + on farm (M2) | 0.37 ± 0.218 | 0.18 ± 0.027 | 0.90 ± 0.129 |  | 0.40 ± 0.074 | 0.17 ± 0.012 | 0.91 ± 0.104 |
|  | FTIR + SNP (M3) | 0.38 ± 0.101 | 0.17 ± 0.002 | 1.06 ± 0.100 |  | 0.41 ± 0.068 | 0.15 ± 0.002 | 1.06 ± 0.087 |
| Urea, mmol/l | FTIR (M1) | 0.42 ± 0.137 | 0.71 ± 0.104 | 1.06 ± 0.027 |  | 0.45 ± 0.092 | 0.69 ± 0.079 | 1.05 ± 0.024 |
|  | FTIR + on farm (M2) | 0.43 ± 0.137 | 0.70 ± 0.131 | 1.05 ± 0.029 |  | 0.46 ± 0.081 | 0.65 ± 0.086 | 1.05 ± 0.024 |
|  | FTIR + SNP (M3) | 0.45 ± 0.178 | 0.68 ± 0.101 | 1.01 ± 0.008 |  | 0.48 ± 0.078 | 0.63 ± 0.087 | 1.01 ± 0.007 |
| *Liver function/hepatic damage* | | | | | | | | |
| AST, U/l | FTIR (M1) | 0.34 ± 0.095 | 19.34 ± 2.16 | 1.14 ± 0.673 |  | 0.37 ± 0.071 | 18.09 ± 1.929 | 1.08 ± 0.099 |
|  | FTIR + on farm (M2) | 0.36 ± 0.120 | 18.52 ± 3.771 | 0.95 ± 0.27 |  | 0.38 ± 0.069 | 17.94 ± 2.955 | 0.96 ± 0.218 |
|  | FTIR + SNP (M3) | 0.37 ± 0.109 | 18.05 ± 3.916 | 1.07 ± 0.424 |  | 0.40 ± 0.065 | 17.49 ± 3.405 | 1.07 ± 0.369 |
| GGT, U/l | FTIR (M1) | 0.39 ± 0.057 | 5.44 ± 0.009 | 0.99 ± 0.179 |  | 0.40 ± 0.054 | 5.60 ± 0.011 | 0.98 ± 0.109 |
|  | FTIR + on farm (M2) | 0.40 ± 0.114 | 5.38 ± 0.268 | 0.95 ± 0.224 |  | 0.43 ± 0.091 | 5.54 ± 0.214 | 0.96 ± 0.121 |
|  | FTIR + SNP (M3) | 0.42 ± 0.061 | 5.30 ± 0.389 | 0.93 ± 0.103 |  | 0.45 ± 0.053 | 5.31 ± 0.338 | 0.97 ± 0.090 |
| PON, U/ml | FTIR (M1) | 0.39 ± 0.151 | 16.09 ± 3.309 | 1.05 ± 0.108 |  | 0.42 ± 0.114 | 16.45 ± 3.111 | 1.05 ± 0.098 |
|  | FTIR + on farm (M2) | 0.42 ± 0.107 | 15.75 ± 0.534 | 1.09 ± 0.068 |  | 0.44 ± 0.091 | 15.31 ± 0.154 | 1.09 ± 0.066 |
|  | FTIR + SNP (M3) | 0.44 ± 0.094 | 15.51 ± 2.283 | 0.98 ± 0.060 |  | 0.46 ± 0.083 | 14.99 ± 1.985 | 0.98 ± 0.057 |
| *Oxidative stress metabolites* | | | | | | | | |
| ROMt, mgH_2_O_2_/100ml | FTIR (M1) | 0.56 ± 0.108 | 1.85 ± 0.147 | 0.97 ± 0.184 |  | 0.61 ± 0.096 | 1.65 ± 0.143 | 1.02 ± 0.084 |
|  | FTIR + on farm (M2) | 0.61 ± 0.084 | 1.82 ± 0.123 | 0.96 ± 0.164 |  | 0.64 ± 0.067 | 1.61 ± 0.111 | 0.97 ± 0.087 |
|  | FTIR + SNP (M3) | 0.64 ± 0.112 | 1.79 ± 0.185 | 1.03 ± 0.193 |  | 0.66 ± 0.097 | 1.58 ± 0.161 | 1.03 ± 0.088 |
| AOPP, µmol/l | FTIR (M1) | 0.32 ± 0.031 | 8.58 ± 0.213 | 0.95 ± 0.095 |  | 0.34 ± 0.019 | 6.94 ± 0.225 | 0.98 ± 0.085 |
|  | FTIR + on farm (M2) | 0.34 ± 0.079 | 7.98 ± 1.247 | 0.94 ± 0.235 |  | 0.37 ± 0.063 | 6.65 ± 1.183 | 0.98 ± 0.19 |
|  | FTIR + SNP (M3) | 0.36 ± 0.021 | 7.75 ± 0.032 | 0.93 ± 0.114 |  | 0.39 ± 0.013 | 6.08 ± 0.028 | 0.99 ± 0.099 |
| FRAP, µmol/l | FTIR (M1) | 0.23 ± 0.039 | 31.94 ± 5.527 | 1.03 ± 0.107 |  | 0.25 ± 0.035 | 32.25 ± 5.001 | 1.02 ± 0.097 |
|  | FTIR + on farm (M2) | 0.24 ± 0.109 | 30.85 ± 3.111 | 0.92 ± 0.099 |  | 0.26 ± 0.087 | 31.75 ± 2.432 | 0.93 ± 0.094 |
|  | FTIR + SNP (M3) | 0.25 ± 0.065 | 30.25 ± 3.471 | 1.15 ± 0.092 |  | 0.27 ± 0.039 | 31.06 ± 3.018 | 1.05 ± 0.082 |
| *Inflammation/innate immunity* | | | | | | | | |
| Ceruloplasmin, µmol/l | FTIR (M1) | 0.52 ± 0.036 | 0.41 ± 0.059 | 1.00 ± 0.053 |  | 0.54 ± 0.032 | 0.38 ± 0.023 | 0.99 ± 0.047 |
|  | FTIR + on farm (M2) | 0.53 ± 0.115 | 0.4 ± 0.025 | 0.96 ± 0.125 |  | 0.56 ± 0.092 | 0.37 ± 0.026 | 0.96 ± 0.092 |
|  | FTIR + SNP (M3) | 0.55 ± 0.083 | 0.39 ± 0.007 | 0.99 ± 0.099 |  | 0.58 ± 0.072 | 0.33 ± 0.006 | 0.99 ± 0.086 |
| PROTt, g/l | FTIR (M1) | 0.69 ± 0.071 | 2.56 ± 0.025 | 1.01 ± 0.035 |  | 0.72 ± 0.069 | 2.26 ± 0.121 | 1.03 ± 0.031 |
|  | FTIR + on farm (M2) | 0.71 ± 0.068 | 2.31 ± 0.506 | 0.99 ± 0.057 |  | 0.73 ± 0.056 | 2.22 ± 0.166 | 1.02 ± 0.055 |
|  | FTIR + SNP (M3) | 0.74 ± 0.032 | 2.28 ± 0.284 | 1.02 ± 0.068 |  | 0.76 ± 0.028 | 2.19 ± 0.247 | 0.99 ± 0.059 |
| Globulins, g/l | FTIR (M1) | 0.71 ± 0.017 | 2.62 ± 0.276 | 0.99 ± 0.081 |  | 0.74 ± 0.015 | 2.33 ± 0.287 | 0.99 ± 0.072 |
|  | FTIR + on farm (M2) | 0.73 ± 0.014 | 2.58 ± 0.593 | 0.97 ± 0.064 |  | 0.75 ± 0.011 | 2.29 ± 0.315 | 0.98 ± 0.051 |
|  | FTIR + SNP (M3) | 0.75 ± 0.029 | 2.49 ± 0.430 | 0.98 ± 0.089 |  | 0.78 ± 0.025 | 2.26 ± 0.374 | 0.98 ± 0.077 |
| *Minerals* | | | | | | | | |
| Calcium, mmol/l | FTIR (M1) | 0.38 ± 0.067 | 0.10 ± 0.007 | 1.03 ± 0.066 |  | 0.40 ± 0.091 | 0.08 ± 0.003 | 1.03 ± 0.065 |
|  | FTIR + on farm (M2) | 0.39 ± 0.069 | 0.09 ± 0.006 | 0.99 ± 0.077 |  | 0.42 ± 0.079 | 0.08 ± 0.002 | 1.05 ± 0.062 |
|  | FTIR + SNP (M3) | 0.41 ± 0.058 | 0.09 ± 0.005 | 1.09 ± 0.031 |  | 0.45 ± 0.043 | 0.08 ± 0.004 | 1.09 ± 0.057 |
| Potassium, mmol/l | FTIR (M1) | 0.47 ± 0.084 | 0.32 ± 0.038 | 0.96 ± 0.137 |  | 0.50 ± 0.075 | 0.28 ± 0.015 | 0.96 ± 0.111 |
|  | FTIR + on farm (M2) | 0.48 ± 0.076 | 0.30 ± 0.020 | 0.96 ± 0.151 |  | 0.52 ± 0.061 | 0.27 ± 0.008 | 0.97 ± 0.102 |
|  | FTIR + SNP (M3) | 0.50 ± 0.143 | 0.29 ± 0.004 | 0.98 ± 0.109 |  | 0.55 ± 0.084 | 0.25 ± 0.003 | 0.98 ± 0.092 |
| Zinc, µmol/l | FTIR (M1) | 0.40 ± 0.159 | 2.15 ± 0.177 | 0.97 ± 0.108 |  | 0.42 ± 0.187 | 1.99 ± 0.166 | 0.97 ± 0.088 |
|  | FTIR + on farm (M2) | 0.42 ± 0.125 | 2.05 ± 0.097 | 0.99 ± 0.105 |  | 0.44 ± 0.164 | 1.86 ± 0.104 | 0.99 ± 0.068 |
|  | FTIR + SNP (M3) | 0.45 ± 0.105 | 1.95 ± 0.269 | 1.05 ± 0.103 |  | 0.47 ± 0.152 | 1.79 ± 0.234 | 0.96 ± 0.062 |

^1^BHBA = β-hydroxybutyric acid; AST = aspartate aminotransferase; GGT = γ-glutamyl transferase; PON = paraoxonase; ROMt = total reactive oxygen metabolites; AOPP = advanced oxidation protein products; FRAP = ferric reducing antioxidant power; PROTt = total proteins. R^2^ – coefficient of determination between the observed and predicted phenotypes in validation set and standard deviation (SD) as the variability measurement of predictive ability; RMSE – root mean squared error.
