# Supplementary material for: Integrating on-farm and genomic information improves the predictive ability of milk infrared prediction of blood indicators of metabolic disorders in dairy cows
Source: Genet Sel Evol. 2023 Apr 3;55:23. doi: 10.1186/s12711-023-00795-1 (PMC10069109; doi:10.1186/s12711-023-00795-1)
Supplement: Supplementary file 2 — Additional file 2: Figure S1. Distribution of the phenotypic values for blood metabolites. Distribution of the phenotypic values for blood metabolites. (a) Energy-related metabolites: BHBA: β-hydroxybutyric acid; GLU: glucose and urea; (b) Liver function/hepatic damage: AST: aspartate aminotransferase, GGT: γ-glutamyl transferase and PON: paraoxonase; (c) Oxidative stress metabolites: AOPP: advanced oxidation protein products, FRAP: ferric reducing antioxidant power, ROMt: total reactive oxygen metabolites; (d) Inflammation/innate immunity: CuCp: ceruloplasmin, GLOB: globulins, PROTt: total proteins; and (e) Minerals: Ca: calcium, K: potassium, and Zn: zinc. Figure S2. oxplot of the phenotypic values for blood metabolites. Boxplot of the phenotypic values for blood metabolites across the six classes of days in milk (DIM). (a) Energy-related metabolites: BHBA: β-hydroxybutyric acid; GLU: glucose and urea; (b) Liver function/hepatic damage: AST: aspartate aminotransferase, GGT: γ-glutamyl transferase and PON: paraoxonase; (c) Oxidative stress metabolites: AOPP: advanced oxidation protein products, FRAP: ferric reducing antioxidant power, ROMt: total reactive oxygen metabolites; (d) Inflammation/innate immunity: CuCp: ceruloplasmin, GLOB: globulins, PROTt: total proteins; and (e) Minerals: Ca: calcium, K: potassium, and Zn: zinc. Class of days in milk (DIM): CL1: less than 60 days; CL 2: from 60 to 120 days; CL 3: from 121 to 180 days; CL 4: from 181 to 240 days; CL 5: from 241 to 300 days; and CL 6: higher than > 300 days. Figure S3. Venn diagrams of the most informative milk FTIR wavelengths for blood metabolites using BayesB and gradient boosting machine (GBM). Venn diagrams showing the unique and shared milk FTIR wavelengths explaining more than 0.8% of the phenotypic variability for BayesB and gradient boosting machine (GBM) for blood metabolites. (a) Energy-related metabolites: BHBA: β-hydroxybutyric acid; GLU: glucose and urea; (b) Liver function/hepatic damage: [file 12711_2023_795_MOESM2_ESM.docx]

**Integrating on-farm and genomic information improves the predictive ability of milk infrared prediction of blood indicators of metabolic disorders in dairy cows**

Lucio FM. Mota^1*^, Diana Giannuzzi^1^, Sara Pegolo^1^, Erminio Trevisi^2,3^, Paolo Ajmone-Marsan^2,3^, Alessio Cecchinato^1^

^1^Department of Agronomy, Food, Natural Resources, Animals and Environment (DAFNAE), University of Padova, 35020, Legnaro (PD), Italy

^2^Department of Animal Science, Food and Nutrition (DIANA) and the Romeo and Enrica Invernizzi Research Center for Sustainable Dairy Production (CREI), Faculty of Agricultural, Food, and Environmental Sciences, Università Cattolica del Sacro Cuore, 29122, Piacenza, Italy

^3^Nutrigenomics and Proteomics Research Center, Università Cattolica del Sacro Cuore, 29122, Piacenza, Italy

*Corresponding author:

Lucio F M Mota, e-mail: [flaviommota.zoo@gmail.com](mailto:flaviommota.zoo@gmail.com)

# Additional File 2

Figure S1 Distribution of phenotypic values of blood metabolites. (a) Energy-related metabolites: BHBA – β-hydroxybutyric acid; GLU – Glucose and Urea; (b) Liver function / hepatic damage: AST = aspartate aminotransferase, GGT = γ-glutamyl transferase and PON = Paraoxonase, (c) Oxidative stress metabolites: AOPP = advanced oxidation protein products, FRAP = ferric reducing antioxidant power, ROMt =total reactive oxygen metabolites, (d) Inflammation/innate immunity: CuCp – Ceruloplasmin, GLOB – Globulins, PROTt – total proteins and (e) Minerals: Ca – Calcium, K – Potassium, and Zn – Zinc.

Figure S2 Boxplot of phenotypic values for blood metabolites across the six classes of days in milk (DIM). (a) Energy-related metabolites: BHBA – β-hydroxybutyric acid; GLU – Glucose and Urea; (b) Liver function / hepatic damage: AST = aspartate aminotransferase, GGT = γ-glutamyl transferase and PON = Paraoxonase, (c) Oxidative stress metabolites: AOPP = advanced oxidation protein products, FRAP = ferric reducing antioxidant power, ROMt =total reactive oxygen metabolites, (d) Inflammation/innate immunity: CuCp – Ceruloplasmin, GLOB – Globulins, PROTt – total proteins and (e) Minerals: Ca – Calcium, K – Potassium, and Zn – Zinc. Class of Days in Milk (DIM): CL1 - less than 60 days; CL 2 - from 60 to 120 days; CL 3 - from 121 to 180 days; CL 4 - from 181 to 240 days; CL 5, - from 241 to 300 days; CL 6 – higher than >300 days.

Figure S3 Venn diagrams showing the unique and shared milk FTIR wavelengths explaining more than 0.8% of the phenotypic variability for Bayesian B (BayesB) and Gradient boosting machine (GBM) for blood metabolites. (a) Energy-related metabolites: BHBA – β-hydroxybutyric acid; GLU – Glucose and Urea; (b) Liver function / hepatic damage: AST = aspartate aminotransferase, GGT = γ-glutamyl transferase and PON = Paraoxonase, (c) Oxidative stress metabolites: AOPP = advanced oxidation protein products, FRAP = ferric reducing antioxidant power, ROMt =total reactive oxygen metabolites, (d) Inflammation/innate immunity: CuCp – Ceruloplasmin, GLOB – Globulins, PROTt – total proteins and (e) Minerals: Ca – Calcium, K – Potassium, and Zn – Zinc.

Figure S4 Pearson correlation between wavelength regions explains more than 0.8% in the GBM approach and blood metabolites in Holstein cows’ population. AOPP = advanced oxidation protein products; AST = aspartate aminotransferase; BHBA – β-hydroxybutyric acid; Ca – Calcium; CuCp – Ceruloplasmin; FRAP = ferric reducing antioxidant power; GGT = γ-glutamyl transferase; GLOB – Globulins; GLU – Glucose; K – Potassium; PON = paraoxonase; PROTt – total proteins; ROMt =total reactive oxygen metabolites; Zn – Zinc.
